# INPP5K limits PtdIns (4,5)P2 accumulation in CD19 microclusters and restrains CD19 co-receptor membrane proximity and BCR-proximal signaling in B cells

**DOI:** 10.3389/fimmu.2026.1934292

**Published:** 2026-09-03

**Authors:** Bastien Moës, Alice Mostafa, Romain Malempré, Olivier Renson, Géraldine Piel, André Matagne, Stéphane Schurmans, Alice Mayer

**Affiliations:** 1 Laboratory of Functional Genetics, GIGA Research Centre, University of Liège, Avenue de l’Hôpital, Liège, Belgium; 2 Laboratory of Enzymology and Protein Folding, Centre for Protein Engineering, InBioS Research Unit, University of Liège, Allée du Août, Liège, Belgium; 3 GIGA Bioinformatics, GIGA Research Centre, University of Liège, Avenue de l’Hôpital, Liège, Belgium; 4 Laboratory of Pharmaceutical Technology and Biopharmacy, Center for Interdisciplinary Research on Medicines, University of Liège, Avenue Hippocrate, Liege, Belgium

**Keywords:** B cells, CD19 siganling molecule, immunology, molecular Biology, phosphoinositides

## Abstract

**Introduction:**

CD19 is a key B-cell co-receptor that amplifies B-cell receptor (BCR) signaling and tunes B-cell activation thresholds. How the conformation of the CD19 cytoplasmic domain is regulated to restrain basal and BCR-induced signaling remains incompletely understood. We investigated whether the phosphoinositide 5-phosphatase INPP5K controls CD19 conformation and CD19-amplified BCR-proximal signaling through local regulation of phosphatidylinositol 4,5-bisphosphate [PtdIns(4,5)P_2_].

**Methods:**

A B-cell-specific *Inpp5k* knock-down (KD) mouse model was generated using CD21-Cre–mediated recombination. CD19–phospholipid interactions were assessed by tryptophan fluorescence with synthetic CD19 peptides and lipid bicelles. CD19 cytoplasmic domain proximity to the plasma membrane was measured by indirect FRET. PtdIns(4,5)P₂ distribution within CD19 microclusters, BCR-proximal signaling, and microcluster dynamics were analyzed by Airyscan microscopy and phospho-flow cytometry. Peripheral B-cell subsets and serum immunoglobulins were quantified by flow cytometry and ELISA.

**Results:**

The CD19 juxtamembrane cytoplasmic domain contains a conserved polybasic region that interacts with acidic phospholipids, including PtdIns(4,5)P_2_. INPP5K KD B cells displayed increased PtdIns(4,5)P_2_ density within CD19 microclusters and a constitutively membrane-proximal CD19 cytoplasmic domain in resting transitional T1/T2 B cells. This was associated with elevated basal phosphorylation of CD19^Y531^, BTK^Y223^ and PLCγ2^Y759^, with preserved inducibility upon BCR engagement, whereas ITAM-proximal components (CD79A, LYN, SYK) were unchanged or reduced. INPP5K deficiency also increased basal CD19 and BCR microcluster formation, impaired CD19–BCR colocalization after stimulation, and altered ezrin phosphorylation and actin organization. These changes coincided with accumulation of T1 B cells, reduced T2, follicular and marginal-zone B cells, and decreased serum IgM and IgG.

**Discussion:**

INPP5K limits local PtdIns(4,5)P_2_ accumulation in CD19 microclusters, thereby restraining membrane association of the CD19 cytoplasmic domain and basal CD19-amplified BCR-proximal signaling. Loss of INPP5K skews the spatial organization and balance of BCR-proximal signaling, with downstream consequences for B-cell microcluster dynamics, peripheral differentiation and humoral output.

## Introduction

The B- (BCR) and T-cell (TCR) antigen receptors are activated only in response to stimuli and have the ability to amplify signals many times (1). The amplification potential requires tight regulation in both resting and stimulated conditions, relying on efficient negative regulatory systems (2).

B cells play an important role in immune responses in both physiological and pathological conditions. The signal activity originating from their antigen-specific receptor is negatively controlled to avoid abnormally increased BCR activity in resting and stimulated conditions that may lead to autoimmunity, cancer or non-autoimmune inflammatory diseases (3–6). Negative control of BCR signaling is mediated by surface molecules such as FcγRIIB/CD32B, CD72, CD5, CD45 and CD22 (7–11) which act through cytoplasmic proteins, and by phosphatases, including the protein tyrosine phosphatases PTPN22 and SHP1/2 and the lipid phosphatases SHIP1 and PTEN (12–16). Mutation, inactivation or dysfunction in one of these negative control elements have been shown to alter B lymphocytes signaling thresholds and responses (7–16).

CD19 is a B-cell-restricted transmembrane glycoprotein that assembles with CD21 (complement receptor 2) and the tetraspanin CD81 into a co-receptor complex which lowers the threshold for B-cell activation and amplifies signaling downstream of the B-cell antigen receptor (BCR) rather than initiating an independent pathway (17–20). Upon BCR engagement, the long CD19 cytoplasmic tail is phosphorylated on conserved tyrosines; phosphorylation of Y531 recruits and activates phosphoinositide 3-kinase (PI3K), which accounts for the majority of BCR-induced PI3K activity and for efficient AKT activation (19, 21, 22). CD19 is also required for the formation and stabilisation of BCR–antigen microclusters, into which it is transiently recruited (23). Through this rheostat function CD19 helps set the balance between antigen-induced responses and tolerance: its loss blunts B-cell responses, whereas even modest CD19 overexpression enhances basal and BCR-induced signaling and predisposes to autoimmunity, yet how CD19 signaling is restrained remains incompletely understood (24–31).

PtdIns (4,5)P2 is a low-abundance but functionally pivotal phosphoinositide concentrated in the inner leaflet of the plasma membrane, where it is non-uniformly distributed in dynamically regulated pools (32–35). Beyond serving as a precursor of second messengers, PtdIns (4,5)P2 and other acidic phospholipids electrostatically engage clusters of basic residues in the juxtamembrane cytoplasmic regions of many receptors, thereby controlling the conformation and accessibility of their signaling motifs. This mechanism has been characterized for the T-cell-receptor CD3 chains, whose cytoplasmic tails are held against the membrane until activation (36–40), and for receptor tyrosine kinases such as the EGFR, whose juxtamembrane segment is regulated by acidic lipids (41, 42). Local changes in acidic-lipid density can therefore switch such juxtamembrane segments between membrane-bound and membrane-released states (43–46).

INPP5K (SKIP) is an inositol polyphosphate 5-phosphatase that dephosphorylates PtdIns (4,5)P2 and PtdIns (3,4,5)P3 and thereby shapes the local phosphoinositide landscape at the plasma membrane and other compartments (47–49). Loss-of-function INPP5K variants cause a congenital muscular dystrophy with cataracts and intellectual disability, and INPP5K regulates membrane and cytoskeletal dynamics in several cell types (50–54). We previously reported that INPP5K inactivation in the haematopoietic compartment markedly perturbs bone-marrow B-cell development through the regulation of IL-7R signaling associate with splenic B cells defects, pointing to a role in BCR-complex signaling (55); how INPP5K controls B-cell signaling at the molecular level had, however, not been defined.

In this work, we identify a novel mechanism that negatively regulates CD19-amplified BCR-proximal signaling by controlling the conformation of the CD19 cytoplasmic domain. This mechanism involves electrostatic interactions between a polybasic region of the CD19 juxtamembrane cytoplasmic domain and plasma-membrane PtdIns (4,5)P2, and we show that inactivation of the PtdIns (4,5)P2 5-phosphatase INPP5K increases PtdIns(4,5)P2 within CD19 microclusters and alters CD19 signaling in resting and BCR-stimulated B cells.

## Materials and methods

### Mice

The construction and validation of our Inpp5k^flox/+^ mice, where exons 2 and 3 of the Inpp5k gene are flanked by LoxP sites, were previously reported (55) (**Supplementary Figure 1A**). Inpp5k^flox/+^ mice were first crossed with CD21-Cre (B6.CgTg(Cr2-cre)3Cgn/J) mice, obtained from the UMR CNRS 7276/INSERM 1262 CRBL, Limoges, France. Then, in order to generate Inpp5k^flox/flox^ CD21-Cre mice, Inpp5k^flox/+^ mice were crossed with Inpp5k^flox/+^ CD21-Cre mice. A mean of 94 ± 4% deletion of exons 2 + 3 was detected by PCR on genomic DNA isolated from Inpp5k^flox/flox^ CD21-Cre (hereafter INPP5K KD) splenic B220^+^ B cells, as compared with Inpp5k^flox/flox^ Cre-negative littermates (hereafter WT) cells (**Supplementary Figure 1B**). Using the Go Taq G2 Hot Start polymerase kit (Promega #M7405), 50 ng of DNA extracted from B220^+^ B cells was used to amplify either the Inpp5k flox or delta allele. To amplify the flox allele, 5’-tct gta gct gga ggg gct aa-3’ and 5’-gct ggc ctc aac tcc tta cg-3’ primers were used; to amplify the delta allele, 5’-tct gta gct gga ggg gct aa-3’ and 5’-tgg gca gta ag gaga gac ca-3’ primers were used. DNA was put onto a 2% agarose gel and a QIAxcel ScreenGel 1.4.0 software was used to quantify DNA amplification. The study involved mice aged between 8 and 16 weeks, with sex randomization conducted and no mice exclusion criteria utilized.For each experiment, the age range and sex of the animals; genotypes were sex- and age-matched littermates All mice were on a C57BL/6 genetic background, were housed in an animal facility with a 12 h light/12 h dark cycle and had free access to food and water throughout the study period. Both sexes of mice were used in the experiments. All mouse studies were authorized by the Animal Care Use and Review Committee of the University of Liège and the protocol number; 2010.

### Human

Peripheral blood from single donor was draw on EDTA tube. The ethics approval/exemption reference ([H-5201]) from the ULiège Ethic Comitee, and that the donor provided written informed consent.

### Reagents and antibodies

BV711 anti-mouse CD45R/B220 (#563892), BUV737 anti-mouse CD24/HAS (#565308), BV421 anti-mouse LY-51/BP1 (#740013), APC anti-mouse IgM (#562032), PE mouse anti-AKT^pT308^ mAb (J1-223.371) (#558275), PE anti-mouse CD23 (B3B4) (#561773), FITC anti-CD21/CD35 (7G6) (#561769), PE mouse anti-PLCγ2 (PY759) (#558490), PE mouse anti-BTK (pY223)/ITK (pY180) (#562753), PE streptavidin (#554061) and PE mouse anti-human CD19 (HIB19) (#560728) antibodies were purchased from BD Biosciences. BV421™ anti-mouse CD19 (#115538), BV711™ anti-mouse CD21/CD35 (CR2/CR1) (#123435), Alexa Fluor^®^ 647 anti-mouse IgM (#406526), PerCP/Cyanine5.5 anti-mouse IgM (#406511), Alexa Fluor^®^ 594 anti-mouse CD19 (#115552) and APC anti-human CD20 (S18015E) (#375505), FITC anti-mouse IgD Antibody (#405703) antibodies were purchased from BioLegend. Anti-phospho-CD19 (Y531) (#STJ90455) and rabbit anti-human INPP5K (#STJ110117) antibodies were purchased from St John’s Laboratory. The anti-phospho-CD19Y531 was validated on mouse spleen cells (**Supplementary Figure 13**). Biotinylated anti-PtdIns(4,5)P2 (#117Z B045) antibody was purchased from Echelon Bioscience. CD19 (F-3) Alexa Fluor^®^ 546 (epitope: DDELAPWEGEGHMGTWMGT) (#sc-373897 AF546) antibody was purchased from Santa Cruz Biotechnology. DiO’/DiOC18(3) (3,3’-Dioctadecyloxacarbocyanine Perchlorate) (#D275), F(ab’)2-donkey anti-rabbit IgG (H+L), PE (#2-4739-81), F(ab’)2-goat anti-mouse IgM (mu), Functional Grade Secondary products (#16-5092-85), Phospho-SYK (Tyr348) PE (#12-9014-42), Goat anti-rabbit IgG (H+L) Highly Cross-Adsorbed Secondary Antibody, Alexa Fluor™ Plus 647, and Alexa Fluor™ 488 (#A32733; #A11034) were purchased from Thermo Fisher Scientific. Anti-phospho-LYN (Y396) rabbit monoclonal was purchased from BosterBio. Phospho-CD79A (Tyr182) rabbit (#5173T) and β-actin (13E5) rabbit mAb (#4790) antibodies were purchased from Cell Signaling Technology. Anti-mouse CD79B antibody (# MAB6814) was purchased from R&D Systems. PE anti-mouse CD43 (leukosialin) (#120431) antibody was purchased from eBioscience. Anti-mouse INPP5K antibodies were produced by Eurogentec and peptide affinity-purified in the Laboratory of Functional Genetics.

### Mouse cell isolation

Spleen was taken from 8- to 16-week-old mice and pressed in a 6 wells plate containing 2 ml of red blood lysis buffer, 3 more ml were added and then incubated 10 min at RT. Lysis was stopped with 10 ml of PBS/10 mM EDTA + 2% FBS and the solution was filtered on a 70 µm membrane and then centrifuged 5 min at 350g. Pellets were resuspended in 1ml of PBS/10 mM EDTA + 2% FBS and alive cells were counted on a Neubauer counting chamber (Hirschmann # 8100103) with Trypan blue (Thermo Fisher Scientific # SV30084.01).

### Blood immunoglobulins quantification

Cardiac puncture was performed on 8- to 16-week-old mice and blood was incubated at RT during at least 30 min and then centrifuged 5 min at 350g. The supernatant was removed in another tube. This step was repeated at least 2 times. Serum levels of IgM and IgG were determined with IgM mouse uncoated ELISA Kit (Plates #88-50470) and IgG (total) mouse uncoated ELISA Kit (Plates # 88-50400-86), both purchased from Invitrogen. Absorbance analysis was performed by Tecan Infinite 200 Pro from LabX.

### Tryptophan fluorescence measurements

Bicelles (q=0.8) were prepared by mixing the long-chain phospholipids POPG (1-palmitoyl-2-oleoyl-phosphatidylglycerol) or POPC (1-palmitoyl-2-oleoyl- phosphatidylcholine) with the short-chain phospholipid DHPC (dihexanoyl- phosphatidylcholine) at a molar ratio of 0.8:1 (POPG/POPC: DHPC). The mixture was dissolved in 20 mM Bis-Tris buffer (pH 6.7) and subjected to multiple freeze-thaw cycles with intermittent vortexing until the lipids were fully dissolved. The CD19 peptide tested in this assay consisted of the mouse CD19 juxtamembrane cytoplasmic peptide (amino acids 311 to 363) to which a neutral, non-polar tryptophan residue was added at the N terminus (WCQRAFILRRKRKRMTDPARRFFKVTPPSGNGTQNQYGNVLSLPTSTSGQAHAQ, obtained from Eurogentec, Belgium). To measure the tryptophan fluorescence emission spectrum of this peptide, 4 µM peptide was incubated with bicelles containing 0–200 µM POPG, POPC, or PtdIns(4,5)P2/POPC lipids. The samples were incubated for 20 min at 20 °C. Intrinsic tryptophan fluorescence emission spectra were recorded at 25 °C using a Varian Cary Eclipse spectrofluorimeter equipped with a Peltier-controlled cell holder and a 1 cm pathlength quartz cuvettes. Samples were prepared in 50 mM Tris-HCl, pH 7.4, 150 mM NaCl, with peptide concentrations of ca. 0.1 mg/ml. Spectra were acquired following excitation at 290 nm.

### Cell phenotyping, BCR cross-linking, facs and western blot analyses

For phenotyping, splenocytes were diluted to 10^6^ cells/ml and then blocked with 100 µl of PBS/10 mM EDTA + FBS 5% + 2.5 ng/ml purified rat anti-mouse CD16/CD32 (BD Biosciences #553141) 20 min at 4 °C. Cells were then centrifuged for 5 min at 350g. Cells were stained with CD19, CD23, CD21 and IgM for 30 min at 4 °C. Each mix was supplemented by 1/1000 Fixable Viability Dye eFluor™ 780 (eBioscience # 65-0865-14).

For *ex-vivo* BCR activation analysis, cells were diluted to 5x10^6^ cells/ml and then blocked with 100 µl of PBS/10 mM EDTA + FBS 5% + 2.5 ng/ml purified rat anti-mouse CD16/CD32 (BD Biosciences #553141) for 20 min at 4 °C. Cells were centrifuged for 5 min at 350g and stained with CD19, CD23, CD21 and IgM antibodies (for BCR cross-linking/activation analysis) in PBS/10 mM EDTA + FBS 5% solution for 30 min at 4 °C. For both condition, antibodies were diluted in Fixable Viability Dye eFluor™ 780 and washed 2 times in PBS/10 mM EDTA +FBS 2%. F(ab’)2-Goat anti-Mouse IgM (μ) (25 µg/ml) was added to cells at 37 °C for 0, 2 and 10 min for BCR activation analysis. The activation was stopped by fixating cells with 2% PFA (Sigma-Aldrich # HT501128) 15 min at 4 °C. Cells were washed 2 times in PBS/10 mM EDTA +FBS 2%, were plasma membrane-permeabilized with Intracellular Fixation & Permeabilization Buffer Set (eBioscience # 88-8824-00), and were stained with anti-AKT^pT308^, anti-PLCγ2^pY759^, anti-CD79A^pY182^, anti-LYN^pY396^, anti-SYK^pY348^, anti-BTK^pY223^ and biotinylated anti-PtdIns(4,5)P2 antibodies for BCR cross-linking analysis. Antibodies were diluted in permeabilization buffer for 50 min at RT and washed 2 times in PBS/10 mM EDTA +FBS 2%. Cells were stained with PE-labeled F(ab’)2-goat anti-rabbit IgG (H+L) secondary antibody (Invitrogen #A10542) or PE streptavidin (BD Bioscience #554061) for 15 min at RT. BD FacsFortessa was used for cell phenotyping. Mean fluorescence intensity (MFI) of specific markers was quantified on B cell subpopulations using FlowJo10.7. Results were analyzed using FlowJo 10.7(Tree Star, Ashland, USA).

In order to analyze INPP5K protein expression in human B cells, human blood was taken on EDTA coated tube (BD Bioscience # 367525) and incubated at RT with 20 mL of red blood lysis buffer for 15 min, then centrifuged 5 min at 350g. The pellet was resuspended in 15 mL of PBS/10 mM EDTA + 2% FBS. This step was repeated at least 2 times. For flow cytometry analysis, blood cells were diluted to 10^6^ cells/ml and then blocked with 100 µl of PBS/10 mM EDTA + FBS 5% + 2.5 ng/ml purified rat anti-mouse CD16/CD32 (BD Biosciences #553141) 20 min at 4 °C. Cells were then centrifuged for 5 min at 350g. Cells were stained with CD19 (BD Biosciences #560728) and CD20 (BioLegend #375505) antibodies for 30 min at 4 °C. Each mix was supplemented by 1/1000 Fixable Viability Dye eFluor™ 780 (eBioscience # 65-0865-14). Cells were fixed with 2% PFA (Sigma-Aldrich # HT501128) 15 min at 4 °C, washed 2 times in PBS/10 mM EDTA +FBS 2% and plasma membrane-permeabilized with Intracellular Fixation & Permeabilization Buffer Set (eBioscience # 88-8824-00). Cells were stained with a rabbit anti-human INPP5K antibody (St John’s Laboratory # STJ110117). Antibody was diluted in permeabilization buffer for 50 min at RT and washed 2 times in PBS/10 mM EDTA +FBS 2%. Cells were then stained with PE-labeled F(ab’)2-goat anti-rabbit IgG (H+L) secondary antibody (Invitrogen #A10542). Mean fluorescence intensity (MFI) of specific markers was quantified on the B cell population using FlowJo. Results were analyzed using FlowJo (Tree Star, Ashland, USA). For Western blot analysis, B cell lysates were prepared by resuspension of isolated human B cells (Miltenyi Biotec # 130-101-638) in 1% Triton X-100 lysis buffer, followed by a 30 min incubation on ice. Lysates were centrifuged at 14,000 rpm for 30 min at 4 °C and the nuclear-free supernatants were subjected to SDS-PAGE on a 10% polyacrylamide gel. Proteins from the gel were electroblotted onto nitrocellulose membranes at 100V for 75 min, followed by 1 h of blocking with 3% BSA (Sigma-Aldrich # A8806) in TBST at RT. The nitrocellulose membranes were then incubated in the presence of the indicated primary antibodies, followed by incubation with HRP-conjugated secondary antibodies (Sigma-Aldrich # A9044). Immunoreactive protein bands were visualized using an ECL reagent (Bio-Rad # 1705061) and autoradiography.

### Immunofluorescence, FRET and Airyscan microscopy analysis

Splenic B cells were purified with mouse CD45R (B220) MicroBeads (#130-049-501). Purified cells were stained with BV421 anti-mouse CD19, Fixable Viability Dye eFluor™ 780, BV711 anti-mouse CD21 and APC anti-mouse IgM antibodies diluted in 100µl of PBS/10 mM EDTA + FBS 5%. FSC-W and FSC-A discrimination was used to exclude doublet cells, and Fixable Viability Dye eFluor™ 780 was used to discriminate between dead and living cells. T1/T2 B cells were sorted as CD19^+^CD21^low^IgM^+^ cells using BD FACSAria III 4L sorter. Nunc™ Lab-Tek™ II Chamber Slide™ System (Thermo Fisher Scientific # 154534PK) was coated with poly-D-lysine hydrobromide (Sigma-Aldrich #P7280) at least 2h at RT. Sorted CD19^+^CD21^low^IgM^+^ cells (50 µl containing 2-4x10^5^ cells) were fixed on slide for 1h at RT. F(ab’)2-goat anti-mouse IgM (μ) (25 µg/ml) was added to cells at 37 °C for 0, 2 and 10 min at 37 °C. Activation was stopped by adding PFA 2% (Sigma-Aldrich # HT501128). For IgM and CD19 microclusters detection, activation was stopped by fixating cells with 2% PFA for 15 min at 4 °C and cells were post-stained with AF647 anti-mouse overnight at 4 °C. Microclusters were defined according to a fluorescence volume threshold (0.3 µm3) that was experimentally defined using Imaris 9 software, in order to exclude background and non-specific fluorescence. Prior to segmentation, Z-stacks were reconstructed with Airyscan (Wiener-filter) deconvolution, which improves resolution and removes shot-noise and out-of-focus artefacts (56); CD19 and IgM/BCR microclusters were then identified automatically in three dimensions in Imaris 9 (Bitplane/Oxford Instruments) as segmented objects with a reconstructed volume greater than 0.3 µm³, a threshold set empirically to exclude background and consistent with reported receptor-microcluster dimensions (23, 57). For phosphoinositides co-localization in microclusters, cells were post-stained with AF594 anti-mouse CD19 and AF647 anti-mouse IgM 30 min at 4 °C. Cells were washed 2 times in PBS/10 mM EDTA +FBS 2%, blocked and permeabilized with a solution containing PIPES 0.65% + NaCl 0.8% + KCl 0.02% + saponin 0.5% + goat serum 10% for 45 min at 4 °C. Cells were stained with a biotynilated PtdIns(4,5)P2 antibody diluted in PIPES 0.65% + NaCl 0.8% + KCl 0.02% + saponin 0.1% + goat serum 10% solution overnight at 4 °C. Cells were washed 3 times in PBS solution and then incubated with 2.5 µg of streptavidin, Alexa Fluor™ 488 conjugate, Alexa Fluor™ 647 conjugate (Thermo Fisher Scientific # S11223; #S21374) for 2h at 4 °C. Cells were washed once in PBS, incubated for 10 min with DAPI 10mM 1/1000 (Thermo Fisher Scientific # D1306) and washed 3 times with dH20. Slide chambers were mounted using ProLong (Invitrogen# P36970). Zeiss LSM880 Airyscan/SIM was used for Z-stacks images acquisition. In Airyscan mode, this system provides a lateral resolution of approximately 120 nm (down to ≈90 nm with joint deconvolution) and an axial resolution of approximately 350 nm, which is sufficient to resolve BCR/CD19 microclusters (reported diameters ≈0.2–1 µm). To ensure valid comparisons, all genotypes and stimulation conditions were fixed and immunostained strictly in parallel with identical reagents and timings, so that any residual effect of chemical fixation applies equally to the samples being compared; PtdIns(4,5)P2 immunostaining was carried out at 4 °C to minimize lateral lipid diffusion during the incubation. Orthogonal reconstructions of the Z-stacks images were obtained with Zen blue and Zen black edition software. For cytoskeletal organization and actin density analysis, cells were fixed with cold methanol at -20 °C for 15 min and wash two times with PBS. Then cells were blocked with PBS + EDTA 10 mM +5% FBS for 30 min at RT and stained with anti β-actin (CST #4970) overnight at 4 °C. Next day, cells were washed 3 times in PBS and stained 2h at 4 °C with AF647/AF488 anti-rabbit (Thermo Fisher Scientific # A32733; #A11034) in PBS + 2%FBS. For FRET, cell activation was stopped by fixating cells with 2% PFA (Sigma-Aldrich # HT501128) for 15 min at 4 °C and were washed 2 times in PBS/10 mM EDTA +FBS 2%. Cells were washed 3 times in PBS, then incubated 10 min with DAPI 10 mM 1/1000 (Thermo Fisher Scientific # D1306) and finally washed 3 times with dH20. Slide chambers were mounted using ProLong (Invitrogen# P36970). Actin filaments were defined after watershed segmentation and MFI measurement was performed on a minimum area of 0.01 µm2 with Fiji software.

For FRET in non-permeabilized condition, cells were blocked in PBS + EDTA 10 mM + 5% FBS and stained and 5 µg of AF488-DiO’ (Thermofisher #D275) in PBS + 10 mM EDTA + 2% FBS 30 min at 4 °C. For FRET in cytoplasmic permeabilized condition, cells were blocked and plasma membrane permeabilized with a solution containing PIPES 0.65% + NaCl 0.8% + KCl 0.02% + saponin 0.5% + goat serum 10% for 20 min at 4 °C and stained with 2.5 µg AF546 anti-mouse CD19 (Santacruz Biotechnology #sc-373897-AF546 immunogen AA 529-543), antibody diluted in PIPES 0.65% + NaCl 0.8% + KCl 0.02% + saponin 0.1% + goat serum 10% solution overnight at 4 °C. Cells were washed 3 times in PBS, then incubated 10 min with DAPI 10 mM 1/1000 (Thermo Fisher Scientific # D1306) and finally washed 3 times with dH20. Slide chambers were mounted using ProLong (Invitrogen# P36970). normalized FRET index was obtained according to a validated calculation that remove fluorescence background (55, 58). For analysis of indirect FRET, compensation for spectral spillover between donor and acceptor channels need to be performed. To this aim, each time, controls conditions were used: cells probed with donor alone (AF488 DIO’), with acceptor alone (AF546 anti CD19) and with both (AF488 DiO’ +AF546 CD19).

### Donor alone (AF488 DiO’)

B = (IDA’)/(IDD).

IDA’= Fluorescence intensity from λdonor excitation and λacceptor emisson.

IDD= Fluorescence intensity from λdonor excitation and λdonor emisson.

Compensation for donor probe (AF488) was established by illuminating the donor probe only sample with the donor excitation wavelength and measuring the background corrected mean intensities within a cell in both donor (IDD) and acceptor (IDA’) channels. DIO’ and AF488 highly cross-adsorbed goat anti-rabbit IgG (H+L) secondary antibody was excited with 488 nm laser line (donor alone) and visualized using the bandpass 500 - 545 + LP 575 Airyscan filter (IDD) or visualized using the 595/40 bandpass + LP 655 Airyscan filter (IDA’).

### Acceptor alone (AF546 anti CD19)

D = (IDA)/(IAA).

IDA= Fluorescence intensity from λdonor excitation and λacceptor emission.

IAA= Fluorescence intensity from λacceptor excitation and λacceptor emission.

Compensation for direct excitation of the acceptor was established using the acceptor probe only (AF546) sample by measuring the background corrected mean intensities within a cell in the acceptor channel using donor excitation wavelength (IDA) and acceptor excitation wavelength (IAA). AF546 cross-adsorbed goat anti-rat IgG (H+L) was excited with 561nm laser line (acceptor alone) and visualized using the bandpass 500 - 545 + LP 575 Airyscan filter (IDA) or using the 595/40 bandpass + LP 655 Airyscan filter (IAA).

### Donor and acceptor (AF488 DiO’ +AF546 anti CD19)

ID= Fluorescence intensity from λdonor excitation and λdonor emission.

IA= Fluorescence intensity from λdonor excitation and λacceptor emission.

IA’= Fluorescence intensity from λacceptor excitation and λacceptor emission.

FRET_corr = IA - B*ID -D*IA’.

FRET_index = FRET_corr/ID.

The samples with both donor probe (AF488) and acceptor probe (AF546) were used to measure FRET. The background corrected intensity in donor (ID) and acceptor channels (IA) were recorded using donor excitation wavelength followed by recording the intensity in the acceptor channel (IA’) using acceptor excitation wavelength. The compensation factors B and D were used to calculate on a pixel-by-pixel basis the corrected FRET intensity (FRETcorr) and the FRET ratio (normalized FRET index) which was reported as a mean value for each analyzed cell and experimental group. For FRET emission (donor + acceptor), only visualization using the 595/40 bandpass + LP 655 Airyscan filter was performed after 561 nm laser line excitation (IA’), but after 488 nm laser line excitation, visualization was performed using the bandpass 500 - 545 + LP 575 Airyscan filter (ID) and using the 595/40 bandpass + LP 655 Airyscan filter (IA).

In order to validate this FRET assay, we defined the maximal and the minimal normalized FRET index using donor and acceptor fluorophores-labeled antibodies directed either against the same target (CD79B: close proximity, positive control) or against 2 targets present in different subcellular compartments (CD79B at the plasma membrane and PAX5 in the nucleus: remoteness, negative control), as previously described (55). For the positive control, cells were blocked, plasma membrane permeabilized, incubated with 2.5 µg of monoclonal rat IgG2A anti-mouse CD79B antibody (R&D Systems # MAB6814 immunogen peptide 29-158) and then incubated with AF546 cross-adsorbed goat anti-rat IgG (H+L) together with AF488 cross-adsorbed goat anti-rat IgG (H+L). normalized FRET index from positive control is defined as the higher limit detection. For FRET negative control, cells were first blocked, plasma membrane permeabilized, incubated with 2.5 µg of monoclonal rat IgG2A anti-mouse CD79B antibody (R&D systems # MAB6814 immunogen peptide 29-158) and then with AF546 cross-adsorbed goat anti-rat IgG (H+L). Secondly, cells were blocked and nuclear-permeabilized with PBS + 5% goat serum + 0.3% triton X100, incubated with monoclonal rabbit IgG anti-mouse PAX5 antibody (CST #12709 immunogen carboxy terminal part) 1/100 and then with AF488 cross-adsorbed goat anti-rabbit IgG (H+L). Antibody dilution solution for nuclear staining used contain PBS + 1% BSA + 0.1% triton X100. normalized FRET index from negative control is defined as the lower limit detection. Zeiss LSM880 Airyscan/SIM was used for Snap images acquisition. Either Zen Zeiss blue or Fiji was used to build images. Fiji was used for script generation, normalized FRET index calculation and image production. The script and methods of statistical analysis are available with this link: https://github.com/Alexh3g0/Indirect_FRET

### Statistical analysis

Respect of the assumptions of normal distribution of residuals and homoscedasticity was verified, and data were presented as mean + SEM as well as individual values, unless otherwise indicated. Data from independent experiments were pooled for analysis in each data panel, unless otherwise indicated. Statistical analyses were performed using Prism 8 (GraphPad Software) or Python script (**Supplementary Text 1**). For nested imaging datasets (FRET, local PtdIns(4,5)P2 and microcluster analyses), cells were pooled from 3 mice per genotype within each experiment, so the individual mouse was not recoverable as an independent unit and the independent experiment was used as the biological replicate (n = 3 independent experiments); per-experiment summaries (medians) are reported and the per-cell distributions are shown as SuperPlots. For flow-cytometry datasets (phospho-flow and B-cell subset quantification), the individual mouse was the biological replicate. Depending on the distribution of the residuals, an unpaired Student’s t-test, a Welch t-test or a Mann-Whitney U test was used as indicated in the figure legends; genotype x stimulation-time effects were evaluated with a factorial (mixed-effects) model rather than multiple independent pairwise tests, and P values were corrected for multiplicity across related comparisons. Effect sizes and 95% confidence intervals are reported where possible. We considered a P-value lower than 0,05 as significant. N.S.: P>0.05; *: P<0.05; **: P<0.01; ***: P<0.001; ****P<0.0001.

## Results

### CD19 contains a conserved juxtamembrane cytoplasmic polybasic region which interacts with PtdIns(4,5)P2 at high concentration

**Figure 1:**
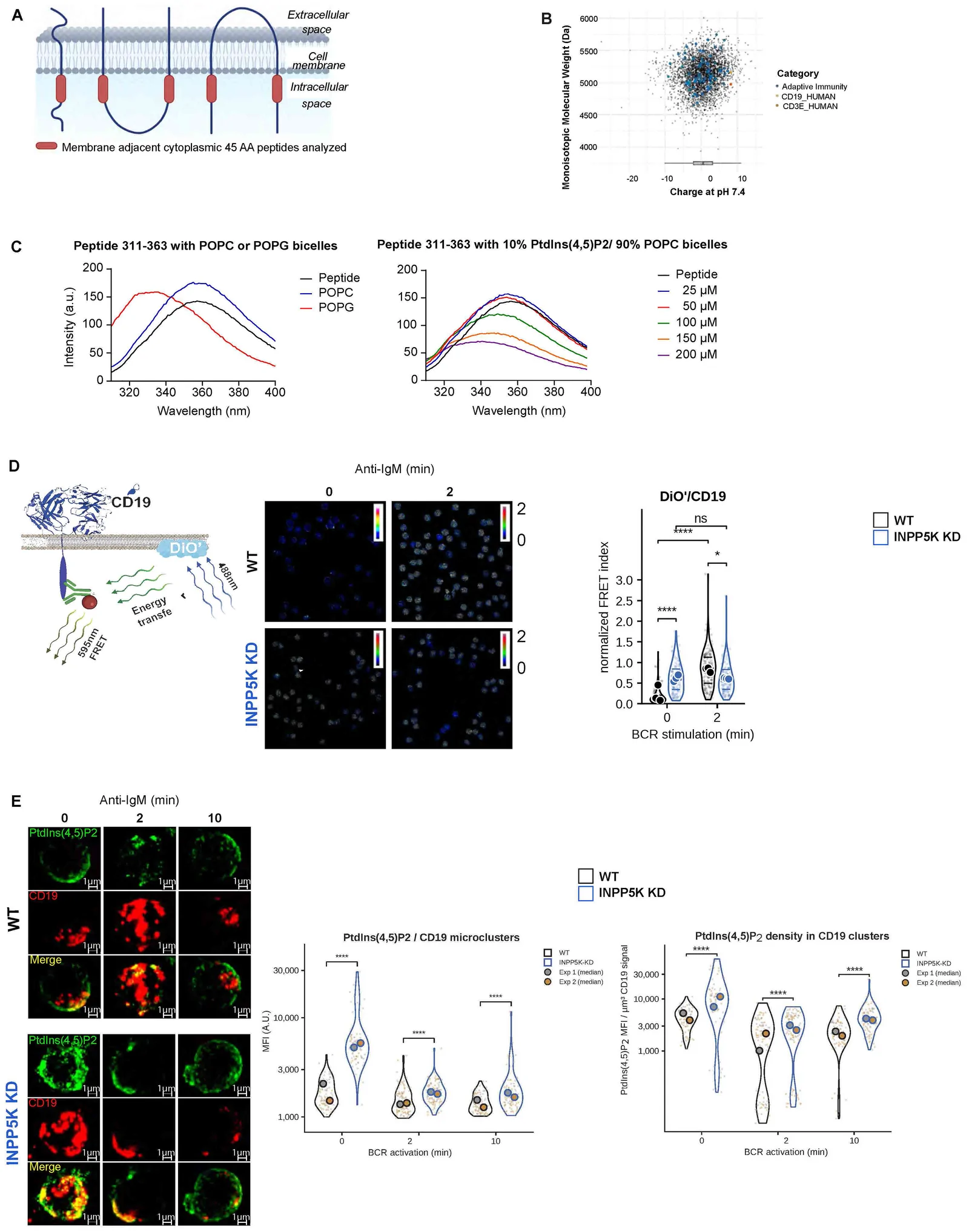
INPP5K and PtdIns(4,5)P2 control the conformation of the CD19 cytoplasmic domain. **(A)** Examples of peptides analyzed in human membrane proteins: only the membrane-adjacent first 45 amino acids of cytoplasmic domains retrieved from the entire human membrane proteome were considered. **(B)** Distribution of charges at pH 7.4 and of monoisotopic molecular weights for cytoplasmic peptides adjacent to plasma membrane in human proteins. Peptide charge was calculated at physiological pH (7.4), using pKa values for native protein conditions. The distribution of these parameters is shown for all peptides (grey dots), with an emphasis on those associated with the “Adaptive Immunity” keyword in the UniProt Knowledgebase (blue dots), as well as specific highlight on peptides from CD3ϵ (red dot) and CD19 (orange dot). The boxplot at the bottom represents the median, 25^th^ and 75^th^ percentiles as well as the 1.5 * inter-quartile range of the charge distribution amongst all peptides. **(C)** Tryptophan fluorescence emission spectra were used to assess the binding of the CD19 cytoplasmic peptide 311–363 to acidic POPG or zwitterionic POPC bicelles (left panel) and to 10% acidic PtdIns(4,5)P2/90% zwitterionic POPC bicelles at different lipid concentrations (right panel). Fluorescence intensity is expressed in arbitrary units (a.u.). The figure shows representative spectra from 3 independent experiments. **(D)** Left image: an indirect FRET strategy was used to analyze the proximity between the fluorescent plasma membrane DiO’ probe and CD19^529–543^ located at the carboxyterminal end of the CD19 cytoplasmic domain in splenic T1/T2 B cells sorted from WT (black columns) and INPP5K KD (blue columns) mice, before (0) and 2 min after BCR cross-linking. Center panels: representative confocal pictures (63X) of energy transfer (normalized FRET index) between AF488- (DIO’) and AF546- (CD19^529-543^) labeled secondary antibody. normalized FRET index was calculated according to the indirect FRET method^30,63^. The rainbow scale represents the energy transfer between the two fluorophores from 0 (blue) to 2 (white). Right graph: the quantitative energy transfer between the two fluorophores-labeled DIO’ and CD19^529-543^ (normalized FRET index) is presented. SuperPlot shows data with the individual experiment as the biological replicate (small dots, individual cells; large dots, per-mouse means; n = 3 mice per genotype per experiment imaged at 0 and 2 min), and the per-cell distributions were unimodal in all conditions (Hartigan’s dip test, all P ≥ 0.48). **(E)** Splenic B cells from WT and INPP5K-KD mice were left unstimulated (0 min) or stimulated with anti-IgM (2 and 10 min), fixed, immunostained for CD19 (red) and PtdIns(4,5)P2 (green), the CD19 antibody was added to cells before plasma membrane-permeabilization while the PtdIns(4,5)P2 antibody was added after membrane permeabilization, and imaged by Airyscan super-resolution microscopy. Left panels: representative confocal microscopy images (100X) of splenic T1/T2 B cells sorted from WT and INPP5K KD mice before (0), and 2 or 10 min after BCR cross-linking. Merge images are also shown. White scale bars: 1 µm. Middle graph: Data are shown as a SuperPlot: small semi-transparent points are individual cells colour-coded by independent experiment; large outlined symbols are the per-experiment medians; violins show the pooled per-cell distribution (log scale). Each data point is the mean PtdIns(4,5)P2 fluorescence intensity (MFI) within CD19-positive microclusters of a single cell. Right graph: Data are shown as a SuperPlot: small points are individual cells colour-coded by independent experiment; large outlined symbols are the per-experiment medians; violins show the pooled per-cell distribution (log_10_ scale. For each cell, PtdIns(4,5)P_2_ fluorescence was normalized to the CD19-positive volume to yield a concentration-like density (MFI per µm³ of CD19 signal) Cells were pooled from 3 mice per genotype per experiment: n = 2 independent experiments (WT black, INPP5K-KD blue). comparison: two-sided Mann–Whitney U test on pooled per-cell values graphs. P values for **(E)** were calculated using a Mann–Whitney U test; for the SuperPlot in **(D, E)**, a linear mixed-effects model with the individual mouse as the biological replicate was used, with Holm correction across the pre-specified contrasts. N.S.: *P*>0.05; *P < 0.05; **P < 0.01; ***P < 0.001; ****P < 0.0001.

Several studies have shown that negatively charged acidic lipids in the inner leaflet of the plasma membrane interact with stretches of positively charged basic amino acids in juxtamembrane cytoplasmic domains of membrane proteins. These ionic interactions play a key role in regulating the conformation and signaling of membrane proteins (36–45, 59, 60). Building on our previous work on the IL-7 receptor (55), the aim of this study was to extend the analysis of the charge at pH 7.4, under physiologic conditions, within juxtamembrane cytoplasmic domains across the entire human membrane proteome (**Figure 1A**; **Supplementary Table 1**; **Supplementary Figure 1**, **Supplementary Material**). The charges of the 3,766 membrane-adjacent, 45 amino acids peptides identified in 2,327 proteins ranged from -27.6 to +14.9, with a median charge of +0.5 and an interquartile range of 5.3 (**Figure 1B**; **Supplementary Table 2**). Our analysis was then refined to focus on the 77 peptides belonging to 65 proteins which play an important role in adaptive immunity, one of the laboratory’s research interests (**Figure 1B**, blue, red and orange dots). CD19 (amino acids 314-358, charge of +8.173, orange dot) and CD3ϵ (amino acids 153-197, charge of +8.152, red dot) were among the peptides with the highest positive charge at pH 7.4 (**Figure 1B**; **Supplementary Table 3**). Since the positive charges in the CD3ϵ juxtamembrane cytoplasmic domain have already been shown to play important roles in phospholipid interaction as well as in regulating its conformation and signaling (37, 44, 45), the potential role of the positively charged residues identified in CD19 was investigated here. Analysis of this juxtamembrane cytoplasmic 45 amino acids CD19 peptide in different species revealed that the sequence is highly conserved and organized in clusters of basic amino acids that are ideal for ionic interaction with acidic lipids (**Supplementary Figure 2**). In order to test whether the polybasic region of CD19 interacts with negatively charged lipids in the inner leaflet of the plasma membrane, we measured intrinsic tryptophan fluorescence emission. This assay assessed the interaction of a synthesized peptide comprising the mouse CD19 polybasic region (amino acids 311 to 363) with lipid bicelles of varying compositions (**Figure 1C**). First, we tested interactions with negatively charged bicelles containing acidic 1-palmitoyl-2-oleoyl-sn-glycero-3-phosphatidylglycerol (POPG) and neutral bicelles containing zwitterionic 1-palmitoyl-2-oleoyl-sn-glycero-3-phosphatidylcholine (POPC). A distinct blue shift in the tryptophan fluorescence spectrum was observed with POPG but not with POPC (**Figure 1C**), indicating that the indole side chain microenvironment changed specifically in the presence of acidic lipid bicelles. Next, we examined the interaction between the same CD19 peptide and bicelles containing 10% acidic PtdIns(4,5)P2 and 90% neutral POPC. PtdIns(4,5)P2 is known to mediate ionic interactions with polybasic regions of membrane proteins and regulate their conformation (37, 38, 42, 44, 46, 55, 59, 60). In some of these studies, expression of the yeast PtdIns(4,5)P2 5-phosphatase Inp54, considered as the ortholog of the mammalian 5-phosphatase INPP5K, served to reduce PtdIns(4,5)P2 level at the plasma membrane and to impair ionic protein-PtdIns(4,5)P2 interactions (19, 44, 46). At low lipid concentrations (25-50 µM), no significant interaction was detected. However, at higher lipid concentrations (100-200 μM), the peptide displayed a blue shift in the tryptophan fluorescence spectrum, demonstrating interaction with PtdIns(4,5)P2 (**Figure 1C**). Although this exceeds the bulk plasma-membrane PtdIns(4,5)P2 concentration (~10–40 µM), PtdIns(4,5)P2 is non-uniformly distributed and forms local, dynamically regulated pools (32–34); the ~3–4-fold local enrichment within CD19 microclusters observed upon INPP5K deletion (**Figure 1E**) would raise the local concentration into this range, indicating that the interaction is achievable at physiological local PtdIns(4,5)P2 densities (35). These results demonstrate that the juxtamembrane cytoplasmic region of CD19 contains a conserved polybasic region which interacts with the 2 acidic phospholipids tested, including PtdIns(4,5)P2, under high lipid concentrations. Importantly, within the CD19 cytoplasmic domain this polybasic region is restricted to the juxtamembrane segment, as no similar sequences were identified further downstream.

### INPP5K and PtdIns(4,5)P2 control the conformation of the CD19 cytoplasmic domain

The PtdIns(4,5)P2 5-phosphatase INPP5K is well expressed in human and mouse CD19^+^ B cells (**Supplementary Figure 3**), and *Inpp5k* inactivation in all hematopoietic cells using *Inpp5k*^flox/flox^ Vav-Cre mice revealed major alterations, notably in bone marrow and splenic B cell development, suggestive of BCR complex signaling alterations (55). Since CD19 is an essential component of the BCR complex, we tested whether *Inpp5k* inactivation specifically in mouse splenic B cells would increase plasma membrane levels of acidic PtdIns(4,5)P2 level. We reasoned that this would strengthen ionic interactions with the polybasic CD19 cytoplasmic domain, thereby altering CD19 conformation and, ultimately, signaling. For this purpose, the *Inpp5k*^flox/flox^ CD21-Cre (INPP5K KD) mouse model was developed, where *Inpp5k* is specifically targeted in mature B cells (**Supplementary Figure 4**). Because INPP5K protein levels were reduced by approximately 50% in B cell subsets from these mice rather than being abolished (**Supplementary Figures 4C, D**), most likely reflecting incomplete Cre-mediated recombination, we refer to this model throughout as an INPP5K knock-down (INPP5K KD). In order to analyze the proximity between the CD19 cytoplasmic domain and the plasma membrane, a likely reflection of the strength of ionic interactions between the CD19 polybasic sequence and plasma membrane acidic phospholipids, including PtdIns(4,5)P2, we used a donor fluorophore-labeled DiO’ membrane probe and an acceptor fluorophore-labeled antibody directed against the C-terminus end of the mouse CD19 cytoplasmic domain (amino acids 529-543) in an indirect FRET assay (**Figure 1D**). Of note, as showed in **Supplementary Figure 5**, we validated this FRET assay, by defining maximal and the minimal normalized FRET index using donor and acceptor fluorophores-labeled antibodies directed either against the same target (CD79B: close proximity, positive control) or against 2 targets present in different subcellular compartments (CD79B at the plasma membrane and PAX5 in the nucleus: remoteness, negative control), as previously described (55). We chose to analyze splenic CD19^+^IgM^+^CD21^low^ T1/T2 B cells in this assay for two reasons: key BCR complex-mediated differentiation events occur at this developmental stage, and CD19 surface expression on these cells was comparable in control (WT) and INPP5K KD mice (**Supplementary Figure 6**). Interestingly, in WT T1/T2 sorted splenic B cells, BCR cross-linking resulted in a marked increase in energy transfer between the excited donor DiO′ membrane fluorophore and the acceptor fluorophore-labeled antibody bound to CD19^529-543^, reflecting a much closer proximity of the CD19 cytoplasmic domain to the plasma membrane when these control B cells are activated (**Figure 1D**). By contrast, the analysis of INPP5K KD T1/T2 B cells in the basal condition revealed a higher energy transfer between the same targets compared with WT T1/T2 B cells, indicating a much closer proximity of the CD19 cytoplasmic domain to the plasma membrane in resting cells when *Inpp5k* is deleted. Of note, energy transfer was not significantly modified after BCR cross-linking of INPP5K KD T1/T2 B cells (**Figure 1D**, left). Because cells were pooled from 3 mice per genotype in each of 3 independent experiments, the independent experiment was used as the biological replicate; BCR engagement increased the membrane proximity of the CD19 cytoplasmic domain in WT B cells but not in INPP5K KD B cells, which were already constitutively membrane-proximal in the resting state, and this pattern was reproducible across the independent experiments. The per-cell distributions were unimodal in every condition (Hartigan’s dip test), consistent with a shift of the whole cell population rather than a mixture of unaffected and affected cells (**Figure 1D**, right). Altogether, our FRET results indicate that the conformation of the CD19 cytoplasmic domain is under control in WT B cells and that INPP5K participates to this control. Indeed, the distance between the polybasic sequence–containing CD19 cytoplasmic domain and the plasma membrane is much shorter in the basal condition in INPP5K KD T1/T2 B cells than in WT T1/T2 B cells, probably due to an increased ionic interaction between the CD19 polybasic sequence and plasma membrane acidic phospholipids. Moreover, in INPP5K KD T1/T2 B cells, this distance is not modified after BCR cross-linking and, at the cell-population level, is intermediate between the basal and BCR-stimulated states of WT T1/T2 B cells; because whole-cell FRET reports a population-averaged proximity, it does not resolve the behavior of individual CD19 molecules. Levels of total cellular PtdIns (4, 5) P2, the main INPP5K substrate, were similar in WT and INPP5K KD T1/T2 B, before and after cross-linking of the BCR (**Supplementary Figure 7**). By contrast, we observed an increased PtdIns(4,5)P2 signal associated with CD19-positive regions in INPP5K KD T1/T2 B cells compared to WT cells, in both resting and BCR-stimulated conditions (**Figure 1E**). In addition, to test whether loss of INPP5K raises the local PtdIns(4,5)P_2_ available to CD19 beyond a simple increase in CD19-positive membrane, we quantified PtdIns(4,5)P_2_ fluorescence within segmented CD19 clusters and normalized it to the CD19-positive volume, yielding a concentration-like density (PtdIns(4,5)P_2_ MFI per µm³ of CD19 signal). This volume-normalized density was consistently higher in INPP5K KD than in WT B cells at rest and after BCR engagement (0, 2 and 10 min), with the same direction of effect in both independent experiments (KD/WT median ratios of 1.3–2.9× at 0 min, 1.2–3.1× at 2 min and 1.8–2.0× at 10 min). Because the increase persists after normalization to CD19 volume, it cannot be attributed merely to a larger CD19-positive compartment in mutant cells: INPP5K deletion raises the molecular density of PtdIns(4,5)P_2_ at CD19 clusters, not only the total local fluorescence. It is noteworthy that the absence of a characteristic background when streptavidin-488 was utilized alone clearly indicates that the observed signal cannot be generated without the primary antibody (**Supplementary Figure S8**). Together with the direct peptide–PtdIns(4,5)P_2_ interaction (**Figure 1C**) and the increased CD19 membrane proximity measured by FRET, this indicates that INPP5K normally restrains a locally concentrated pool of PtdIns(4,5)P_2_ engaged by the CD19 juxtamembrane polybasic motif. Increased PtdIns(4,5)P2 levels markedly enhance ionic interactions and proximity between the CD19 cytoplasmic domain and the plasma membrane in the basal condition. In the BCR-stimulated condition, they are associated with an abnormal, constitutively membrane-proximal state of the CD19 cytoplasmic domain that, at the cell-population level, is intermediate between the basal and BCR-stimulated states observed in WT T1/T2 B cells.

### Constitutive CD19-amplified BCR-proximal signaling in INPP5K KD T1/T2 B cells

**Figure 2:**
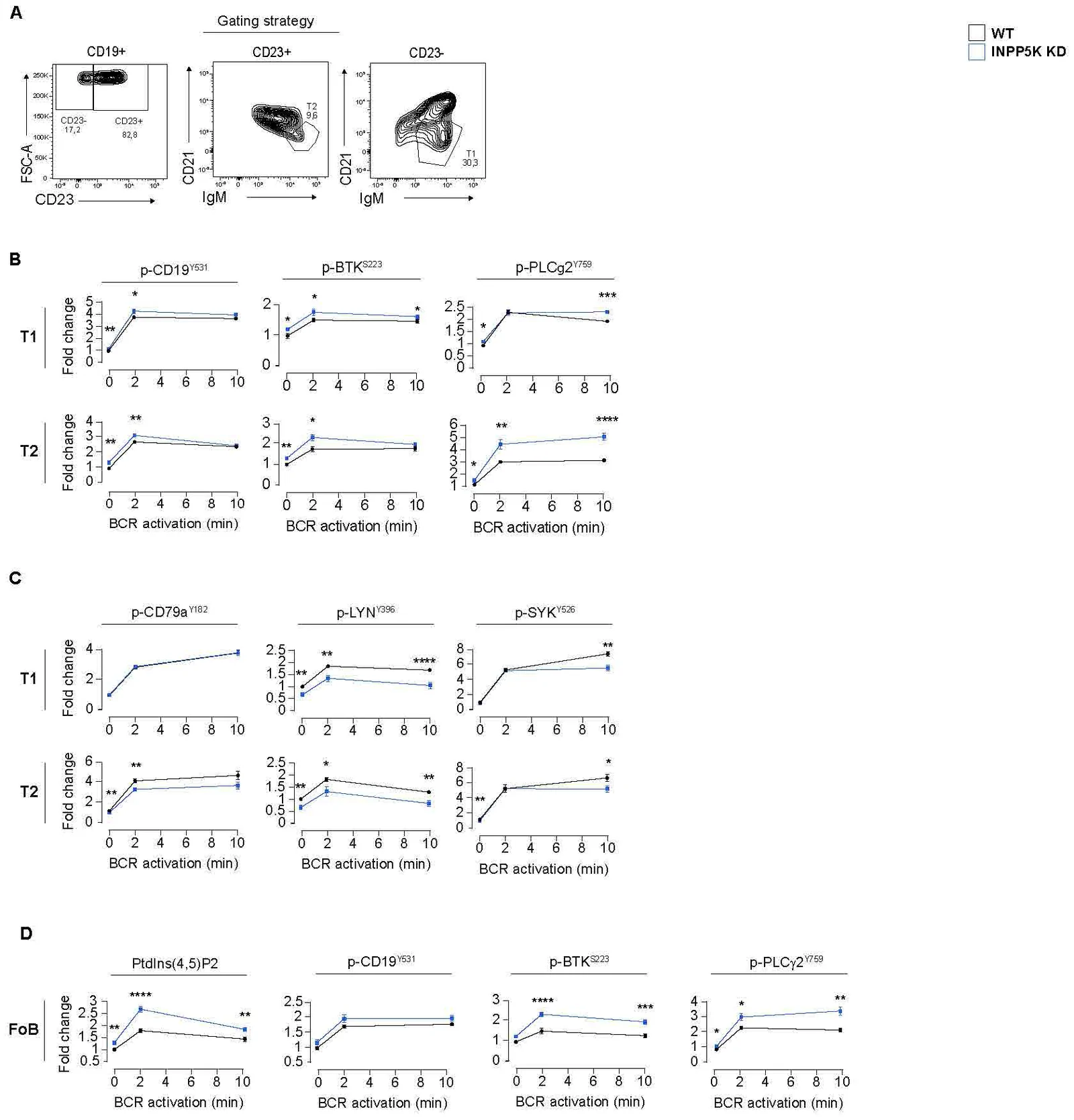
Skewing of BCR-proximal signaling toward the CD19-amplified arm in splenic INPP5K KD transitional B cells. **(A)** The staining strategy used to identify splenic T1 and T2 B cells is shown. **(B, C)**. Flow cytometry analysis of p-CD19^Y531^, p-BTK^Y223^ and p-PLCγ2^Y759^(B, CD19-amplified BCR-proximal components)and of p-CD79A^Y182^, p-LYN^Y396^ and p-SYK^Y348^ (C, ITAM-proximal BCR components: CD79A/LYN/SYK) in plasma membrane-permeabilized T1 and T2 cells isolated from WT (black lines) and INPP5K KD (blue lines) spleen, before (0), and 2 or 10 minutes after BCR cross-linking. Quantified expression normalized to WT MFI at t = 0 (before BCR cross-linking) are presented; n= 6–12 mice per group. Data are pooled from 2–4 independent experiments. **(D)**. Splenic FO B cells from WT (black) and INPP5K KD (blue) mice were analyzed by phospho-flow cytometry before (0) and 2 or 10 min after BCR cross-linking (F(ab’)2 anti-IgM). PtdIns(4,5)P2 levels and phosphorylation of p-CD19^Y531^, p-BTK^Y223^ and p-PLCγ2^Y759^ are expressed as fold change relative to the WT resting condition (mean ± SEM; n = 5–6 mice per genotype). *P* values were calculated using Mann–Whitney U test. N.S.: *P*>0.05; *P < 0.05; **P < 0.01; ***P < 0.001; ****P < 0.0001.

Changes in membrane proteins conformation due to altered ionic interactions with plasma membrane acidic phospholipids, including PtdIns(4,5)P2, are known to positively or negatively impact downstream signaling (36–45, 55, 59). In line with these conformational results, one signaling defect in INPP5K KD T1 and T2 B cells, compared with WT cells, was a significantly increased CD19^Y531^ phosphorylation in the basal condition, one of the earliest events that normally follows physiological BCR complex activation (**Figures 2A, B**). This specific CD19^Y531^ phosphorylation typically enables PI3K binding and the activation of its downstream signaling pathway (19, 21, 61). Concomitantly, downstream of this constitutive CD19 phosphorylation, a constitutive phosphorylation of BTK^Y223^ and PLCγ2^Y759^ were also detected in INPP5K KD T1 and T2 B cells, compared to WT B cells (**Figure 2B**). After BCR stimulation, phosphorylation of CD19, BTK, PLCγ2 and AKT was increased in INPP5K KD B cells, compared with WT B cells (**Figure 2B**; **Supplementary Figure 8**). By contrast, BCR/surface Ig signaling was affected differently in INPP5K KD B cells (**Figures 2A, C**). Indeed, phosphorylation of CD79a^Y182^, LYN^Y396^ and SYK^Y348^, three of the earliest events after surface immunoglobulin cross-linking and activation, was either normal or significantly decreased in INPP5K KD B cells, as compared with WT B cells (**Figure 2C**). Altogether, our results reveal a skewing between the activation of BCR-proximal signaling toward the CD19-amplified arm in INPP5K KD B cells, compared with WT B cells, with increased basal and BCR-stimulated activation of CD19, BTK and PLCγ2.

### Constitutive CD19 and BCR microclusters formation and colocalization defects in INPP5K KD T1/T2 B cells

**Figure 3:**
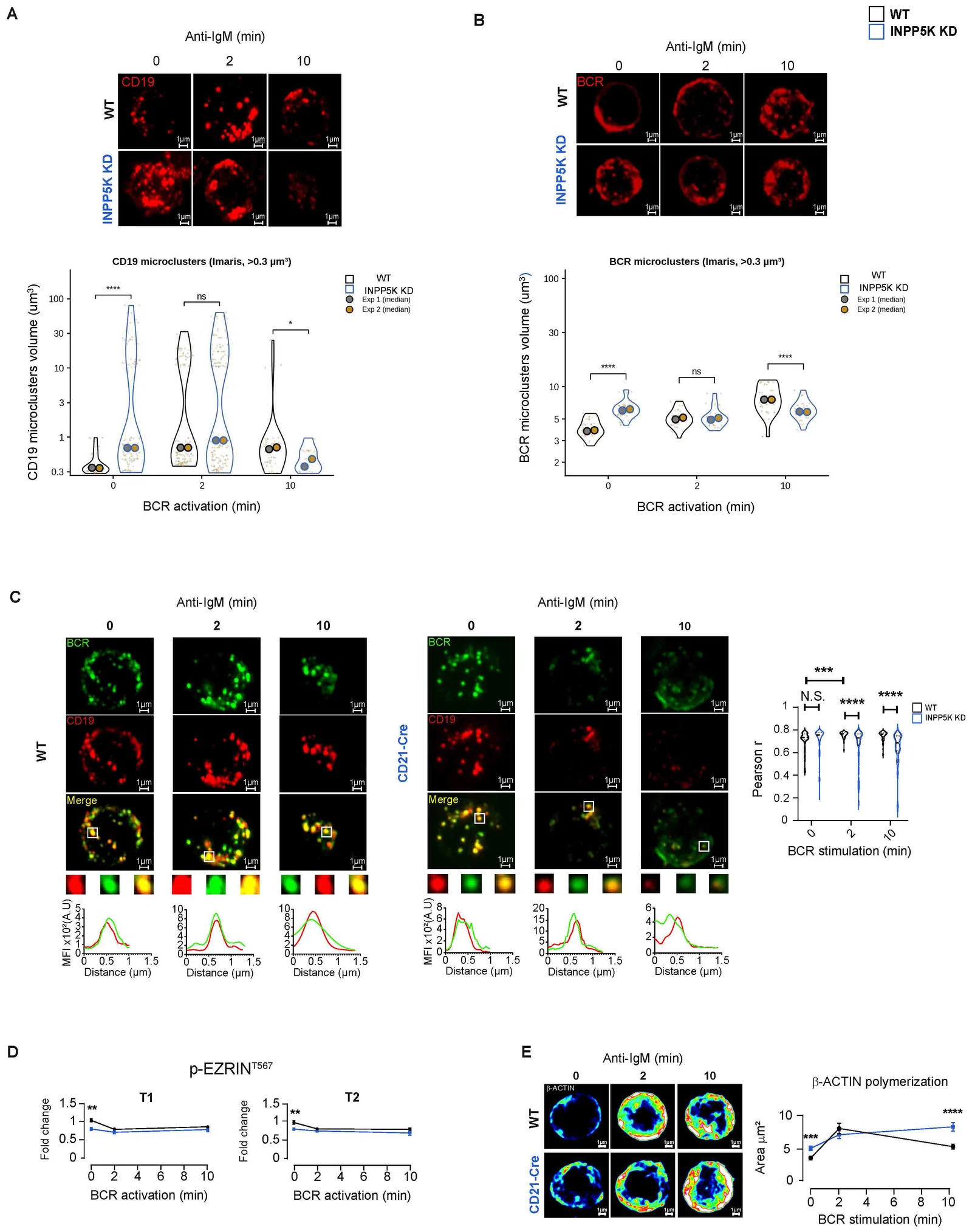
Constitutive CD19 and BCR microclusters formation and co-localization defects in INPP5K KD T1/T2 B cells. **(A, B)** Left images: representative airyscan pictures (100X) of CD19 **(A)** and BCR **(B)** microclusters in splenic CD19^+^IgM^+^CD21^low^ (T1/T2) sorted B cells from WT and INPP5K KD mice before (0), and 2 or 10 min after anti-IgM (25µg/ml) addition. White scale bar: 1µm. Right Graphs: CD19 **(A)** and BCR **(B)** microclusters volume (> 0.3 µm^3^) was analyzed by airyscan microscopy in splenic CD19^+^CD21^low^IgM^+^ (T1/T2) sorted B cells from WT (black lines) and CD21-Cre (blue lines) mice after staining with AF-594 anti-CD19 **(A)** or AF-488 anti-IgM **(B)** antibodies, before (0), and 2 or 10 min after addition of anti-IgM (25µg/ml). Because the yield of sorted T1/T2 cells was limited, cells from three mice per genotype were pooled within each of two independent experiments; these data are therefore shown with the experiment as the biological replicate (SuperPlot: small dots, individual microclusters colour-coded by experiment; large dots, per-experiment medians; n = 2 independent experiments), and the phenotype was reproducible across both experiments. Results represent microclusters ± SEM (n=50–100 cells from 6 mice per group). **(C)** Left images: representative airyscan pictures (100X) of BCR microclusters (green), CD19 microclusters (red) and BCR/CD19 co-localization (yellow) in splenic CD19+IgM+CD21low (T1/T2) sorted B cells from WT and INPP5K KD mice, before (0), and 2 or 10 min after anti-IgM (25µg/ml) addition. White scale bar: 1µm. Left graphs: superposition of BCR (green) and CD19 (red) signals along a distance within a specified microcluster defined by the white square in the corresponding pictures, in splenic CD19+IgM+CD21low (T1/T2) sorted B cells from WT (black lines) and INPP5K KD (blue lines) mice, before (0), and 2 or 10 min after anti-IgM (25µg/ml) addition. Right graph: Pearson correlation factor of the superposition of BCR and CD19 signals along a distance within the above specified microcluster in splenic CD19+CD21lowIgM+ (T1/T2) sorted B cells from WT (black lines) and INPP5K KD (blue lines) mice, before (0) and 2 or 10 min after anti-IgM (25µg/ml) addition. Results represent means Pearson correlation factor ± SEM (n=75–100 microclusters of 20–30 cells from 6 mice per group). **(D)** Flow cytometry analysis of p-EZRIN^T567^ in plasma membrane-permeabilized T1 and T2 cells sorted from WT (black lines) and INPP5K KD (blue lines) spleen, before (0), 2 or 10 minutes after BCR cross-linking. Quantified expression normalized to WT MFI at t=0 (before BCR cross-linking) are presented; n= 6 mice per group. Data are pooled from 2 independent experiments. **(E)** Left images: representative confocal pictures (100X) of β-ACTIN staining in CD19^+^IgM^+^CD21^low^ (T1/T2) sorted B cells from WT and INPP5K KD mice, before (0) and 2 or 10 min after anti-IgM (25µg/ml) addition. White scale bar: 1µm. Right graph: β-ACTIN MFI was analyzed by confocal microscopy in CD19^+^CD23^low^IgM^+^ (T1/T2) B cells from WT (black line) and CD21-Cre (blue line) mice before (0), and 2 or 10 min after addition of anti-IgM (25µg/ml). Results represent area (µm^2^) of β-ACTIN staining after watershed segmentation relative to WT before activation (t=0) ± SEM (n=30–50 cells from 3 mice per group). *P* values were calculated using Mann–Whitney U test. N.S.: *P*>0.05; *P < 0.05; **P < 0.01; ***P < 0.001; ****P < 0.0001. .

BCR and CD19 microclusters formation, growth and colocalization are essential for robust initiation and sustained BCR complex signaling. Multiple factors positively or negatively affect microclusters formation, growth and colocalization. These factors include PtdIns(3,4,5)P3 and PtdIns(4,5)P2 relative levels and the activity of their metabolizing enzymes (eg: PI3K, PTEN and INPP5B), as well as FcγRIIb and CD19 B cell coreceptors signaling (62–66). Since the altered CD19 cytoplasmic domain conformation observed in both basal and BCR-stimulated INPP5K KD T1/T2 B cells is associated with increased CD19-amplified BCR-proximal signaling, we analyzed the formation and colocalization of CD19 and BCR microclusters in these cells (**Figure 3**). As expected from the literature, both BCR and CD19 microclusters (defined as segmented objects with a volume higher than 0.3 µm3) increased over time in WT T1/T2 B cells in response to BCR cross-linking (**Figures 3A, B**). Interestingly, CD19 microclusters volume were bigger at the surface of INPP5K KD T1/T2 B cells as compared with WT B cells in the resting state (**Figure 3A**). To a lesser extent, similar results were observed for the formation of BCR microclusters (**Figure 3B**). Two minutes after BCR cross-linking, increased colocalization of BCR and CD19 microclusters was detected in WT T1/T2 B cells (**Figure 3C**), in agreement with the literature (66). However, in INPP5K KD T1/T2 B cells, CD19 and BCR microclusters progressively dissociated, resulting in a significantly lower Pearson correlation coefficient and reduced colocalization at 2 and 10 min after BCR cross-linking compared with WT B cells (**Figure 3C**). Mechanistically, microclusters formation at the B cell surface normally requires BCR stimulation, transient dephosphorylation/deactivation of ezrin (**Figure 3D**), reducing the linking of cortical actin to plasma membrane proteins and allowing mobility and clustering of BCR complex components, and finally actin cytoskeleton reorganization (**Figure 3E**). In INPP5K KD T1/T2 B cells, low levels of phospho-EZRIN (p-EZRIN) were already detected in the basal condition, as compared with WT B cells, close to the lowest levels observed in WT B cells, and 2 min after BCR stimulation (**Figure 3D**). These low p-EZRIN levels were associated with an increased polymerized β-ACTIN network before and 10 min after BCR stimulation (**Figure 3E**). Our results indicate that in resting INPP5K KD B cells, the actin network is constitutively dissociated from plasma membrane proteins, allowing mobility and clustering of the BCR complex components in the absence of BCR stimulation. It is noteworthy here that constitutive PLCγ2 activation (**Figure 2B**) and ezrin dephosphorylation are both observed in resting INPP5K KD T1/T2 B cells. BCR ligation is known to induce threonine dephosphorylation of ezrin and its detachment from actin, transiently uncoupling the plasma membrane from the cortical cytoskeleton (67).

Altogether, these results indicate that INPP5K deletion is associated with constitutive CD19 and BCR microclusters formation, probably secondary to constitutive PLCγ2 activation and ezrin dephosphorylation. The CD19-BCR microclusters colocalization defect may explain the CD19-BCR signaling imbalance described above in INPP5K KD B cells. It is noteworthy here that within these CD19 microclusters, phosphorylation of SYK was significantly increased in T1/T2 INPP5K KD B cells 2 min after BCR cross-linking, as compared with WT B cells, whereas not when total cellular SYK phosphorylation was analyzed (**Supplementary Figure 10**; **Figure 2C**). Thus, our results highlight the association between the altered CD19 cytoplasmic domain conformation/CD19 signaling and the constitutive formation of CD19 microclusters.

To determine whether these signaling alterations extend beyond the transitional compartment, which represents only a minor fraction of the splenic B cell pool, we analyzed mature follicular (FO) B cells from WT and INPP5K KD mice (**Figure 2D**). FO B cells from INPP5K KD mice also showed enhanced CD19-amplified BCR-proximal signaling, with significantly increased phosphorylation of PLCγ2 (Y759) at rest and after BCR cross-linking, and of BTK (Y223) after cross-linking. Total cellular PtdIns(4,5)P2 was likewise increased at all time points in these cells. However, the signature differed from that of transitional cells (T1/T2) in two important respects: CD19 (Y531) phosphorylation was not constitutively increased in FO B cells, in contrast to its constitutive elevation in resting and BCR-stimulated T1/T2 cells; and total cellular PtdIns(4,5)P2 was increased at all time points in FO B cells, whereas in T1/T2 cells total PtdIns(4,5)P2 was unchanged and only the PtdIns(4,5)P2 associated with CD19 microclusters was elevated (**Figure 2D**; **Supplementary Figure 7**; **Figure 1E**). These observations suggest that the specific PtdIns(4,5)P2-dependent control of CD19 cytoplasmic-domain conformation characterized here is most prominent at the T1/T2 stage, while follicular B cells display related but mechanistically distinct signaling alterations. Because follicular B cells derive from the transitional pool, we cannot formally distinguish signaling defects intrinsic to follicular cells from those inherited as a downstream consequence of the abnormalities already present in their T1/T2 precursors.

### Altered peripheral B cell differentiation and decreased serum immunoglobulin levels in INPP5K KD mice

**Figure 4:**
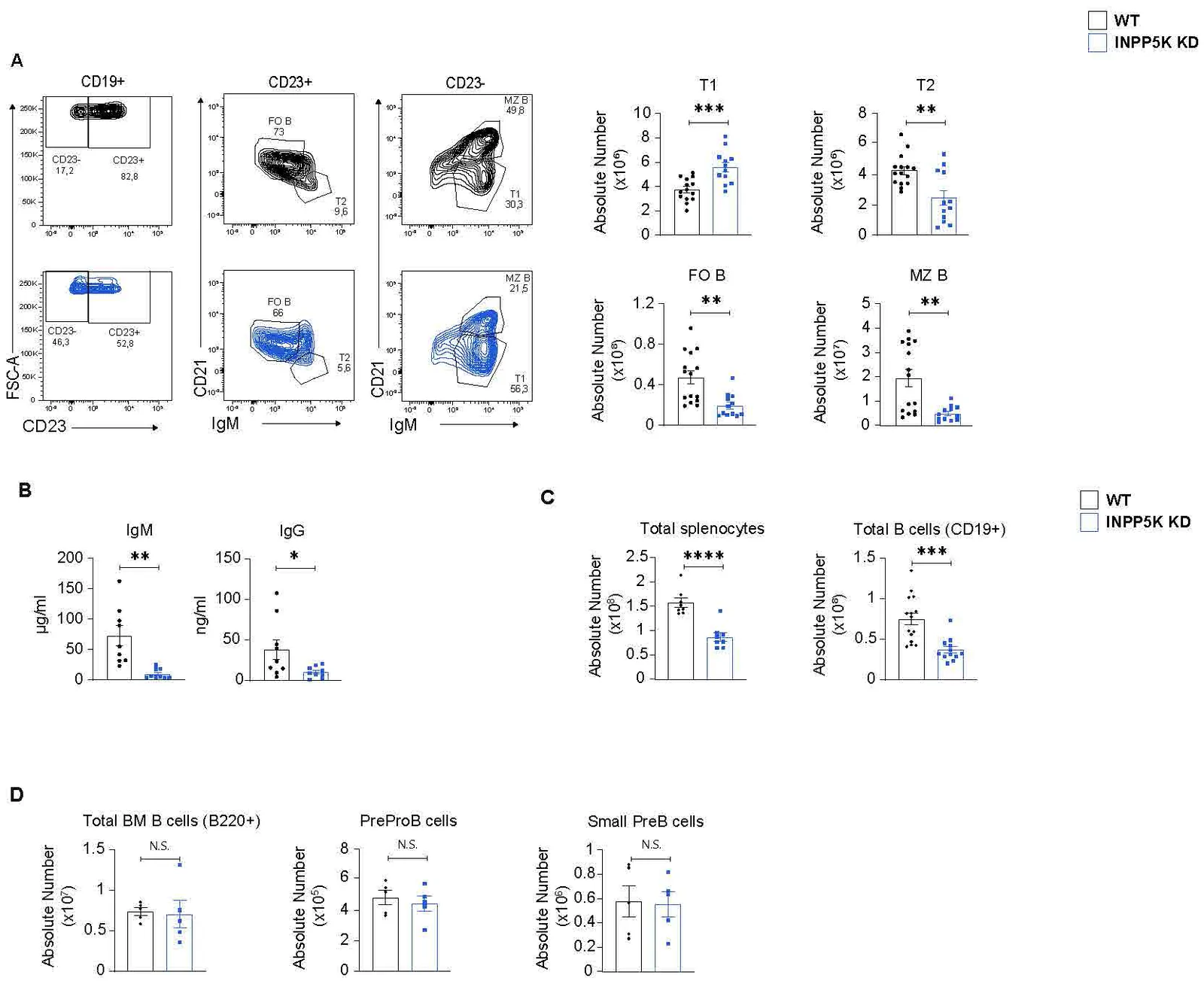
Altered peripheral B cell differentiation and decreased serum immunoglobulin levels in INPP5K KD mice. **(A)** Flow cytometry analysis of WT and INPP5K KD splenic B cell development. Upper panels: staining strategy to identify the different splenic B cell subsets: Transitional 1 (T1, CD19^+^CD23^-^CD21^-^IgM^+^), Transitional 2 (T2, CD19^+^CD23^+^CD21^-^IgM^+^), Follicular (FO B, CD19^+^CD23^+^CD21^Int^IgM^int^) and Marginal Zone (MZ B, CD19^+^CD23^-^CD21^+^IgM^+^). Lower graphs: B cell numbers in each splenic subset of WT (black columns and dots) and INPP5K KD (blue columns and dots) mice. Results represent individual mice and means ± SEM. n=12–15 mice per group. Data are pooled from 3 independent experiments. Similar results were obtained by using an alternative staining strategy (**Supplementary Figure 11**). **(B)** ELISA analysis of blood WT (black columns and dots) and INPP5K KD (blue columns and dots) IgM and IgG immunoglobulins concentrations. Results represent individual mice and means ± SEM; n=8–9 mice per group. Data are pooled from 2 independent experiments. *P* values were calculated using Mann–Whitney U test. N.S.: *P*>0.05; *P < 0.05; **P < 0.01; ***P < 0.001; ****P < 0.0001. **(C, D)** Absolute splenic cellularity and bone-marrow B-cell development in WT and INPP5K KD mice. Total splenocyte and total splenic B-cell (CD19^+^) numbers (left) **(C)**, and bone-marrow total B cells (B220^+^) and early precursors (Pre-Pro-B/Fraction A (B220^+^CD43^+^BP-1^-^HSA^-^)); Small Pre-B/Fraction D (B220^+^CD43^-^IgM^-^IgD-)) (right) **(D)**. Bars, mean ± SEM; each symbol = one mouse; Mann–Whitney U test; *P<0.05, **P<0.01, ***P<0.001; ns, not significant.

Signaling through the BCR complex is required for splenic B lymphocytes maturation. Defects in components of the BCR signalosome may result in defects in peripheral B cell differentiation. Analysis of B cell subsets in the spleen of INPP5K KD mice revealed a significant increase of T1 B cell number associated with a decrease of T2, FO and MZ B cells, compared to WT mice (**Figure 4A**). An alternative staining strategy revealed similar results (**Supplementary Figure 11**). Finally, a marked reduction of both IgM and IgG levels was found in the serum of unchallenged INPP5K KD mice, compared with WT mice, a possible consequence of the decreased FO and MZ B cell numbers (**Figure 4B**). To localize the developmental origin of the peripheral phenotype, we quantified absolute splenic cellularity and bone-marrow B-cell development. Total splenocyte and total splenic B-cell (CD19^+^) numbers were significantly reduced in INPP5K KD mice (**Figure 4C**). By contrast, bone-marrow B-cell development was comparable between genotypes: total bone-marrow B cells (B220^+^) and the early Pre-Pro-B (Fraction A) and Small Pre-B (Fraction D) compartments did not differ (**Figure 4D**). Together with the accumulation of T1 and the reduction of T2, follicular and marginal-zone cells (**Figure 4A**), these data indicate that the contracted peripheral B-cell compartment of INPP5K KD mice arises from a block at the peripheral transitional checkpoint rather than from impaired central B-cell output — consistent with the mature-B-cell-restricted CD21-Cre deletion and with the constitutive CD19-amplified signaling described above.

Thes results also indicate that *Inpp5k* deletion in splenic B cells is associated with defects in B cell differentiation and immunoglobulin production.

## Discussion

Among the different peptides that emerged from our membrane proteome-wide bioinformatic study, we chose to further analyze the potential interactions between the positively charged amino acids region present in the CD19 juxtamembrane cytoplasmic domain and negatively charged lipids in the inner leaflet of the plasma membrane. Indeed, this CD19 basic cytoplasmic region is highly conserved in different species and is restricted to the juxtamembrane segment of the CD19 cytoplasmic domain. Moreover, this type of positively charged juxtamembrane region was not detected in the cytoplasmic domains of the other components of the BCR complex (**Supplementary Table 4**), offering thus the possibility to analyze the specific role of the CD19 basic cytoplasmic region in the wider physiological context of BCR complex activation and functions.

We discovered that INPP5K deletion in B cells is associated with increased PtdIns(4,5)P2 levels in CD19 positive regions and with an abnormal, high activation-prone conformation of the CD19 cytoplasmic domain. The likely consequences of this high activation-prone CD19 structural alteration are increased CD19 signaling and CD19 microclusters formation in resting and BCR-stimulated B cells from INPP5K KD mice. In other words, we pinpointed for the first time how important the regulation of CD19 cytoplasmic domain conformation is to control signaling, and may be differentiation and immunoglobulin production, in B cells. Because BTK, PLCγ2 and AKT are shared, BCR-proximal effectors rather than CD19-exclusive readouts, we interpret their altered activation as evidence of CD19-amplified BCR-proximal signaling rather than of a separate, CD19-autonomous pathway. CD19 is a well-established amplifier of BCR-proximal signaling: upon BCR ligation it is phosphorylated by Lyn and recruits phosphoinositide 3-kinase (PI3K), PLCγ2 and other effectors, establishing a Src-family kinase amplification loop that lowers B-cell activation thresholds (20, 68), and it is required for the formation and stabilisation of BCR–antigen microclusters into which it is transiently recruited (57). In this framework, increased local PtdIns(4,5)P_2_ at CD19 clusters could promote CD19 co-receptor engagement and the local recruitment of CD19-associated PI3K; the resulting signaling could be spatially concentrated at the co-receptor rather than uniformly amplified across the receptor.

This view reconciles two observations that would otherwise appear discordant. First, the increase in AKT phosphorylation despite unchanged whole-cell PtdIns(3,4,5)P_3_ is expected if PI3K output and AKT engagement are restricted to CD19 microclusters: CD19 accounts for the majority of BCR-induced PI3K activity and is necessary for efficient AKT activation (22), so a locally elevated but spatially confined PtdIns (3–5)P_3_ pool can drive AKT at the cluster without measurably shifting the bulk cellular PtdIns (3–5)P_3_, which is dominated by the much larger non-cluster membrane. Second, the reduced or unchanged CD79A, LYN and SYK phosphorylation argues against global hyperactivation of the ITAM-proximal arm and is instead consistent with a redistribution, or skewing, of signaling toward the CD19 co-receptor arm; notably, amplification through the CD19/Lyn loop can occur while SYK phosphorylation remains unchanged (68). We therefore present the phenotype as a change in the spatial organisation and balance of BCR-proximal signaling, not as activation of an independent CD19 pathway.

We also emphasize that the proposed downstream sequence — local PtdIns(4,5)P_2_ enrichment promoting PLCγ2 activity and ezrin-dependent cytoskeletal remodeling to stabilize CD19 microclusters — is advanced as a mechanistic hypothesis consistent with the present observations, not as an established causal chain. Direct perturbation of PLCγ2 or ezrin, and a demonstration of CD19-restricted PI3K recruitment, would be required to establish causality and are important goals for future work; we now state this explicitly as a limitation. A further methodological limitation concerns the detection of PtdIns(4,5)P2. Because a biotinylated anti-PtdIns(4,5)P2 antibody revealed with tetrameric streptavidin was used, the detection reagent itself may promote some clustering of the lipid. The identical staining procedure applied to all genotypes and conditions renders any such cross-linking-induced reorganisation systematic rather than genotype-specific, and the streptavidin-only control confirms that no signal is generated in the absence of the primary antibody (**Supplementary Figure 8**); nevertheless, minimising this artefact would be preferable to controlling for it, and the use of fluorescent monovalent binders would be the method of choice for future nanoscale analyses of PtdIns(4,5)P2 distribution.

In our molecular model, INPP5K hydrolyzes PtdIns(4,5)P2 in CD19 microclusters, preventing high ionic interactions and so high proximity between the CD19 cytoplasmic domain and the inner leaflet of the plasma membrane, particularly in the basal condition. This specific function seems unique to INPP5K. Thus, in the physiological context, a greater distance is observed between the CD19 cytoplasmic domain and the membrane in the basal condition, what seems necessary to prevent a constitutive CD19 signaling activation. In the BCR-stimulated condition, PtdIns(4,5)P2 level in CD19 microclusters of INPP5K KD B cells is markedly decreased compared to the resting condition, but still significantly increased compared to BCR-stimulated WT B cells. This PtdIns(4,5)P2 level seems still sufficient to freeze CD19 in the high activation-prone conformation in BCR-stimulated INPP5K KD B cells. In BCR-stimulated WT B cells, despite lower PtdIns(4,5)P2 level in the CD19 microclusters, a closer proximity between the CD19 cytoplasmic domain and the plasma membrane is observed, as compared to INPP5K KD B cells. In the BCR-stimulated condition, change in the conformation of the CD19 extracellular domain, may be in combination with other charged factors than PtdIns(4,5)P2, like Ca^2+^ ions and other membrane phospholipids, could also participate in the regulation of the distance between the CD19 cytoplasmic domain and the membrane. However, we cannot definitively exclude that the elevated PtdIns(4,5)P2 level observed in INPP5K KD CD19 microclusters is also involved in some other mechanisms, beside the new CD19 conformational mechanism presented here.

Our results also illustrate the complexity of the control of BCR/CD19 microcluster formation by phosphoinositides and phosphoinositide-regulating enzymes, including PTEN, INPP5B and now INPP5K phosphatases. The non-redundant role of INPP5K and INPP5B in this control is noteworthy: INPP5K preferentially regulates PtdIns(4,5)P2 level and microcluster formation in the basal state, while INPP5B exclusively regulates the same parameters after BCR activation (62).

The normal CD19 cytoplasmic conformation seems necessary to prevent CD19 signaling overactivation, which may lead to autoimmunity. In this context, it is noteworthy that significantly decreased *INPP5K* mRNA levels are observed in naïve B cells isolated from highly active SLE patients, as compared with inactive SLE patients (69), suggesting a role for INPP5K in SLE disease activity, as long as the B cell populations are not skewed between the 2 patients groups (**Supplementary Figure 12**).

A 20% B cell surface overexpression of CD19 in humans and a 15-29% overexpression in transgenic mice are associated with increased basal and BCR-stimulated CD19 signaling, as well as with autoimmunity features (27–31). Altogether with our data in INPP5K KD B cells, these results confirm that cell surface CD19 functions as a general rheostat for defining constitutive and antigen receptor-induced B lymphocyte signaling thresholds. The mechanism leading to the constitutive CD19 phosphorylation and signaling when CD19 is overexpressed seems to involve an enhanced LYN phosphorylation/activity due to the increased CD19 density at the B cell surface, followed by a processive amplification of CD19 phosphorylation (20, 70). In INPP5K KD B cells, total cellular level of phosphorylated LYN^Y396^ was decreased compared with WT B cells, in apparent contradiction with the constitutively increased CD19^Y531^ phosphorylation/activation observed in these cells. Nevertheless, similarly to what was observed for PtdIns (4, 5)P2 levels above, it remains possible that basal LYN phosphorylation or activity is enhanced specifically in CD19 microclusters of INPP5K KD B cells, compared to WT B cells. In addition, the abnormal, high activation-prone conformation of the CD19 cytoplasmic domain seen in resting and BCR-stimulated INPP5K KD B cells could unmask important tyrosines, like CD19 Y^531^, and make them much more accessible for LYN, SYK and/or PI3K binding and/or phosphorylation. In the specific case of PI3K, both increased binding to the CD19 cytoplasmic domain and a greater proximity of this domain to the plasma membrane, where the PI3K substrate is present, could be involved in CD19 signaling activation. In this context, Tedder and collaborators demonstrated that approximately 2% of cellular LYN is constitutively associated with CD19 in resting B cells (70). BCR cross-linking increases LYN’s binding to CD19 by ~2-fold. In contrast, BCR cross-linking was required to induce a significant association between PI3K and CD19, with the amount of PI3K associated with CD19 increasing more than 10-fold after BCR stimulation (20, 70).

In conclusion, we uncovered a novel and significant mechanism regulating CD19-amplified BCR-proximal signaling in B cells, beside the previously reported protein-protein interactions with the CD19 extracellular domain and the phosphorylation of specific tyrosines within the CD19 cytoplasmic region. This active process likely involves the 5-phosphatase INPP5K, which dephosphorylates its substrate PtdIns(4,5)P2 within CD19 microclusters and controls the conformation of the CD19 cytoplasmic domain. Finally, our study is the first to describe the role of INPP5K specifically in CD19 microclusters and signaling, extending thus the role of this phosphatase out of the endoplasmic reticulum, the lysosome and the cytoplasm, and out of endothelial, renal, neuronal and skeletal muscle cells (47–54).

## Data Availability

The code used in this study is available on Zenodo: https://doi.org/10.5281/zenodo.16936017 under the GNU Affero General Public License v3.0 or later.
