# Supplementary material for: INPP5K limits PtdIns (4,5)P2 accumulation in CD19 microclusters and restrains CD19 co-receptor membrane proximity and BCR-proximal signaling in B cells
Source: Front Immunol. 2026 Sep 3;17:1934292. doi: 10.3389/fimmu.2026.1934292 (PMC13581894; doi:10.3389/fimmu.2026.1934292)
Supplement: Supplementary file 1 [file DataSheet1.pdf]

Lipid-dependent control of CD19 conformation by INPP5K shapes B cell signaling

Supplementary Material:

In order to extend the analysis of the charge within membrane-adjacent, 45 amino acids peptides of cytoplasmic domains across the entire human membrane proteome, the freely accessible UniProt KnowledgeBase database containing millions of annotated protein sequences was used (1). Indeed, the UniProtKB programmatic access interface (API) and a custom Python3 script served to download data, including amino acid sequence, length, topological domains as well as transmembrane and intramembrane domain positions, for all documented human membrane-associated proteins (request criteria: organism_id:9606 = Homo sapiens, keyword:KW-0472 = membrane). Of the 66,734 entries matching these criteria, proteins lacking positional information for their cytoplasmic domain were filtered out, resulting in 3,895 membrane-associated proteins with at least one documented cytoplasmic region (supplementary Table 1). To extract the 45 amino acid peptides closest to the membrane from cytoplasmic domains, a Python-based bioinformatic pipeline was designed (supplementary Fig. 3). This pipeline first identified the position of all cytoplasmic regions from the main isoform of each membrane protein using topological domain information. Next, these domains were filtered to retain only those adjacent to transmembrane or intramembrane regions. Peptides from cytoplasmic domains shorter than 45 amino acids were excluded, while those exactly 45 amino acids long were kept. For longer domains, we extracted the first and eventually last 45 amino acids, retaining only peptides adjacent to membrane regions. To determine the charge of the resulting 3,766 peptides at pH 7.4 under native conditions, the Peptide Tool from ProtPI was used via their web interface (https://www.protpi.ch/Calculator/PeptideTool).

Supplementary Tables:

Supplementary Table 1:

Detailed information on human membrane-associated proteins with documented cytoplasmic domains, retrieved from the UniProt Knowledgebase

Table1_Uniprot_hum-KWmembrane_Filtered-cytoplasmic_20240829.xlsx

Supplementary Table 2:

Characteristics of selected cytoplasmic human peptides.

Table2_cytoplasmic_peptides_characteristics.xlsx

Supplementary Table 3:

Top 6 human peptides which play an important role in adaptive immunity, ranked by highest positive charge at pH 7.4.

UniProt ID	Entry Name	Start	Stop	Charge at pH 7.4	Amino Acid Sequence
O14672	ADA10	694	738	+8.89	GFIKICSVHTPSSNPKLPPPKPLPGT LKRRRPPQPIQQPQRQRPR
P06127	CD5	403	447	+8.56	KKLVKKFRQKKQRQWIGPTGMNQN MSFHRNHTATVRSHAENPTAS
P15391	CD19	314	358	+8.17	QRALVLRRKRKRMTDPTRRFFKVTP PPGSGPQNQYGNVLSLPTPT

P07766	CD3ε	153	197	+8.15	KNRKAKAKPVTRGAGAGGRQRGQ NKERPPPVPNPDYEPIRKGQRD
O75509	TNR21	371	415	+6.52	RKSSRTLKKGPRQDPSAIVEKAGLK KSMTPTQNREKWIYYCNGHG
Q9UIB8	SLAF5	247	291	+6.38	RLFKRRQGRIFPEGSCLNTFTKNPY AASKKTIYTYIMASRNTQPA

Supplementary Table 4:

Cytoplasmic domains of the main BCR complex components in T1, T2, FO and MZ B cells: name, amino acids sequence and number, as well as the charge at pH 7.4 of the entire cytoplasmic domain (1-end) and of the first 45 amino acids adjacent to the plasma membrane (1-45).

Name	Sequence of the cytoplasmic domain in the mouse (number of AA)	Charge at pH 7.4	
		1- end	1- 45
surface IgM	KVK (3)	-	-
CD79A/Igα	RKRWQNEKFGVDMPDDYEDENLYEGLNLDDCSM YEDISRGLQGTYQDVGNLHIGDAQLEKP (**61**)	-9.5	-7.5
CD79B/Igβ	DKDDGKAGMEEDHTYEGLNIDQTATYEDIVTLRTG EVKWSVGEHPGQE (48)	-9.0	-7.1
CD19	CQRAFILRRKRKRMTDPARRFFKVTPPSGNGTQN QYGNVLSLPTSTSGQAHAQRWAAGLGSVPGSYG NPRIQVQDTGAQSHETGLEEEGEAYEEPDSEEGS EFYENDSNLGQDQVSQDGSGYENPEDEPMGPEE	-30.4	+8.5

	EDSFSNAESYENADEELAQPVGRMMDFLSPHGS<br>AWDPSREASSLGSQSYEDMRGILYAAPQLHSIQS<br>GPSHEEDADSYENMDKSDDLEPAWEGEGHMGT<br>WGTT (237)		
CD21	KHRESNYYTKTRPKEGALHLETREVYSIDPYNPAS<br>(35)		+1.0
CD81	N-terminus: MGVEGCTKCIKY (12)<br>C-terminus: CCGIRNSSVY (10)		+0.1<br>+0.2
CD45	IGIDAMLEGLEAEGKVDVYGYVVKLRRQRCLMVQ<br>VEAQYILIHQALVEYNQFGETEVNLSELHSCLHNM<br>KKRDPPSDPSPLEAEYQRLPSYRSWRTQHIGNQE<br>ENKKKNRNSNVVPYDFNRVPLKHELEMSKESEPE<br>SDESSDDDSDSEETSKYINASFVMSYWKPEMMIA<br>AQGPLKETIGDFWQMIFQRKVKVIVMLTELVNGDQ<br>EVCAQYWGEGKQTYGDMEVEMKDTNRASAYTLR<br>TFELRHSKRKEPRTVYQYQCTTWKGEELPAEPKD<br>LVSMIQDLKQKLPKASPEGMKYHKHASILVHCRDG<br>SQQTGLFCALFNLLESAETEDVVDVFQVVKSLRKA<br>RPGVVCSYEQYQFLYDIIASIYPAQNGQVKKTNSQ<br>DKIEFHNEVDGGKQDANCVRPDGPLNKAQEDSR<br>GVGTPEPTNSAEEPEHAANGSASPAPTQSS (441)	-20.8	-1.5

CD32/ FcγRIIB	KKKQVPALPGNPDHREMGETLPEEVGEYRQPSG GSVPVSPGPPSGLEPTSSSPYNPPDLEEAAKTEA ENTITYSLLKHPEALDEETEHDYQNHI (94)	-11	-1.1
CD72	LQVSRQFQEGTRIWEATNSSLQQQLREKISQLGQ KEVELQKARKELISSQDTLQEKQRTHEDAEQQLQ ACQAERAKTKENLKTEEERRRDLDQRLTSTRETL RRFFSDSSDTCCPCGWIPYQERCFYISHTLGSLEE SQKYCTSLSSKLAAFDEPSKYYYEYLSDAPQVSLP SGLEELLDRSKSYWIQMSKKWRQDSDSQSRHCV RIKTYYQKWERTISKCAELHPCICESEAFRFPDGIN LN (243)	-5.4	-0.1
CD31	RKAKAKQKPVEMSRPAAPLLNSNSEKISEPSVEAN SHYGYDDVSGNDAVKPINQNKDPQNMDVEYTEV EVSSLEPHQALGTRATETVYSEIRKVDPNLMENRY SVSTHVTHTTLKMHP (118)	-3.1	+2.9

Supplementary Figures legends:

supplementary Figure 1

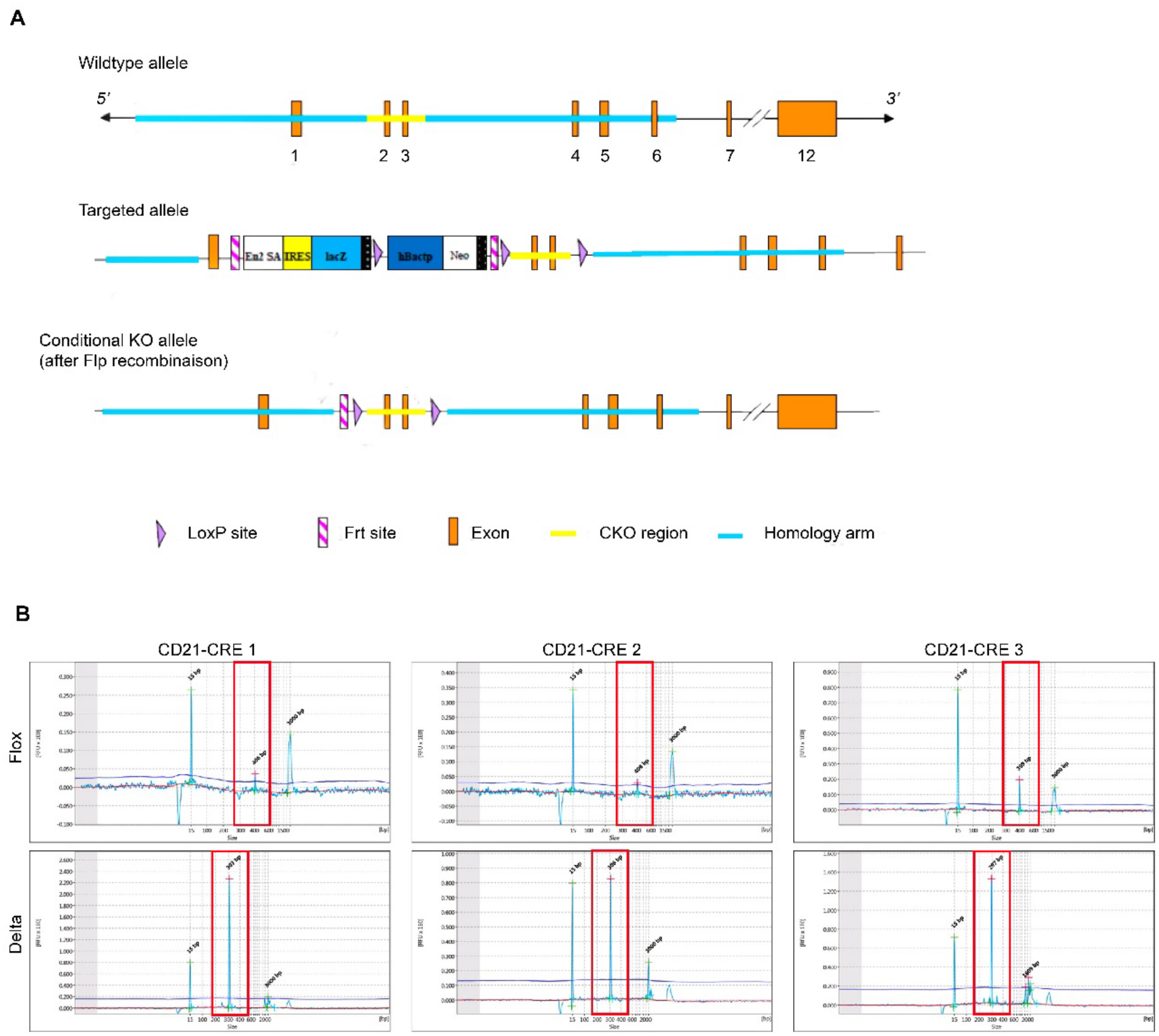

	CD21-CRE 1	CD21-CRE 2	CD21-CRE 3
Delta presence	2,29	0,83	1,33
Flox presence	0,029	0,035	0,192
Total absorbance	2,319	0,865	1,522
Deletion percentage	99	96	87
Mean	94		
SD	4		

Supplementary Figure 1: The *Inpp5k*$^{flox/flox}$ CD21-Cre (CD21-CRE) mouse. A. The mouse wild type, targeted and conditional knockout (KO) *Inpp5k* alleles are represented. Orange boxes denote exons; the blue and the yellow lines represent the homology arms of the targeting vector and the conditional knockout (CKO) region, respectively; purple

triangles are LoxP sites and pink and white striped boxes are Frt sites. The targeting vector contains 2 cassettes: the first with an En2 splice acceptor (SA) site followed by an internal ribosome entry site (IRES) and a lacZ gene, the second with a human β actin promoter (hBactp) followed by the neomycin phosphotransferase gene (Neo). The CKO allele is represented after Flp recombination. **B**. In order to assess the efficiency of the CD21-CRE recombinase, the *Inpp5K* flox and delta alleles were amplified in 2 separate PCR, using genomic DNA isolated from WT and CD21-CRE splenic B220$^+$ B cells as template. Agarose gel and QIAxcel ScreenGel 1.4.0 software were used to quantify flox (±400bp) and delta (±300bp) amplicons.

supplementary Figure 2

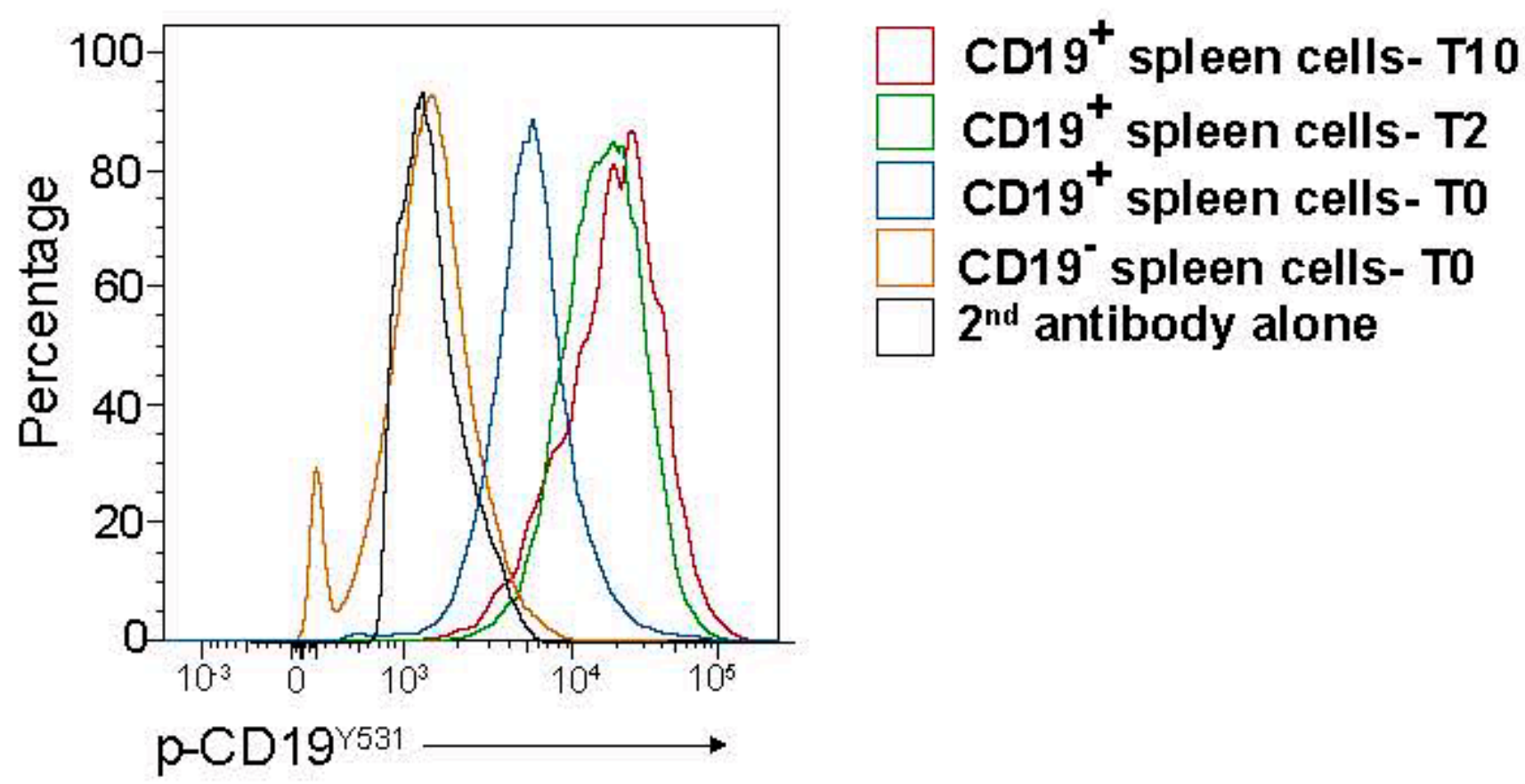

Supplementary Figure 2: Validation of the p-CD19^{Y131} antibody in mouse spleen cells. Representative facs plot of p-CD19^{Y131} in mouse plasma membrane-permeabilized spleen cells: 2nd antibody alone (black line), mouse CD19^{-} spleen cells (orange line), mouse CD19^{+} spleen cells at steady state (blue line), mouse CD19^{+} spleen cells 2 min after BCR cross-linking (green line) and mouse CD19^{+} spleen cells 10 min after BCR cross-linking (red line)

supplementary Figure 3

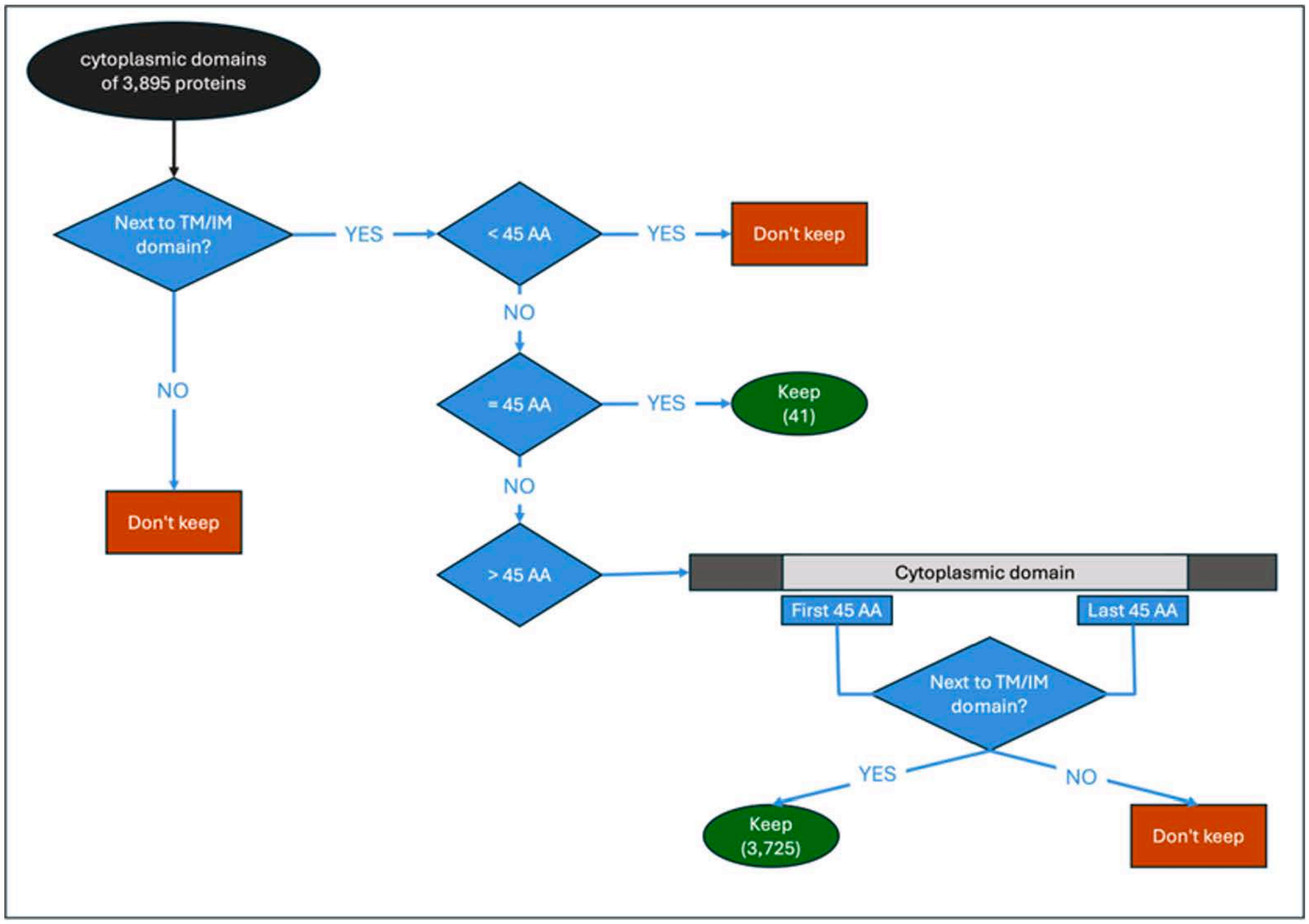

Supplementary Figure 3: Schematic representation of the algorithm used to extract the first 45 amino acids from cytoplasmic domains adjacent to documented transmembrane or intramembrane regions. For each cytoplasmic domain, we first determined whether it was located next to a transmembrane or intramembrane domain. If not, the domain was excluded. If adjacent, the domain length was analyzed: domains shorter than 45 amino acids were excluded, domains exactly 45 amino acids long were retained (41 peptides). For domains longer than 45 amino acids, we extracted the first and last 45 amino acids, retaining only those fragments adjacent to transmembrane or intramembrane domains (3,725 peptides).

supplementary Figure 4

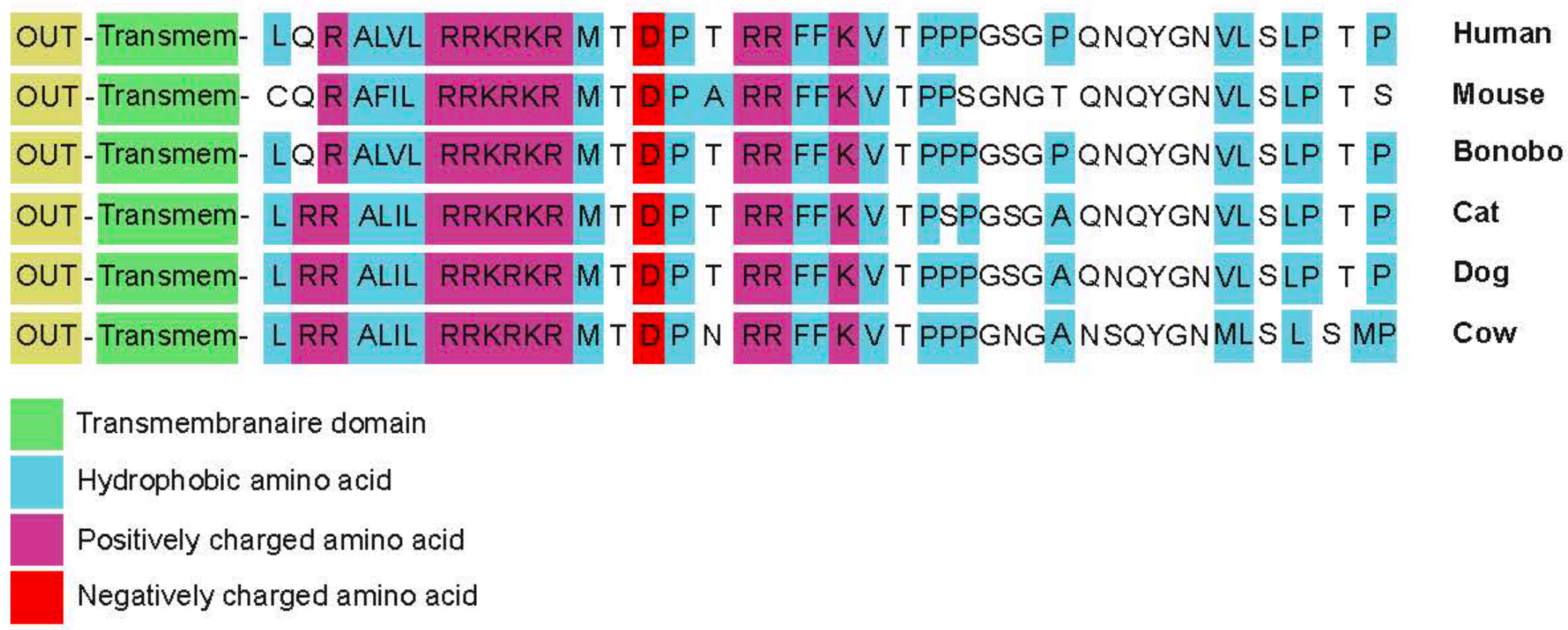

Supplementary Figure 4: Amino acids sequence alignment of the CD19 juxtamembrane cytoplasmic regions from different species. Presence of a conserved positively-charged polybasic amino acid sequence in the juxtamembrane region of the CD19 cytoplasmic domain in different species. The transmembrane region (green), hydrophobic (blue), positively (purple) and negatively (red) charged amino acids are represented. The domain is organized in clusters of basic amino acids that are separated by a suitable distance and by hydrophobic residues, and contains very few acidic residues, all parameters favoring ionic interactions with negatively charged acidic lipids, including PtdIns(4,5)P2.

supplementary Figure 5

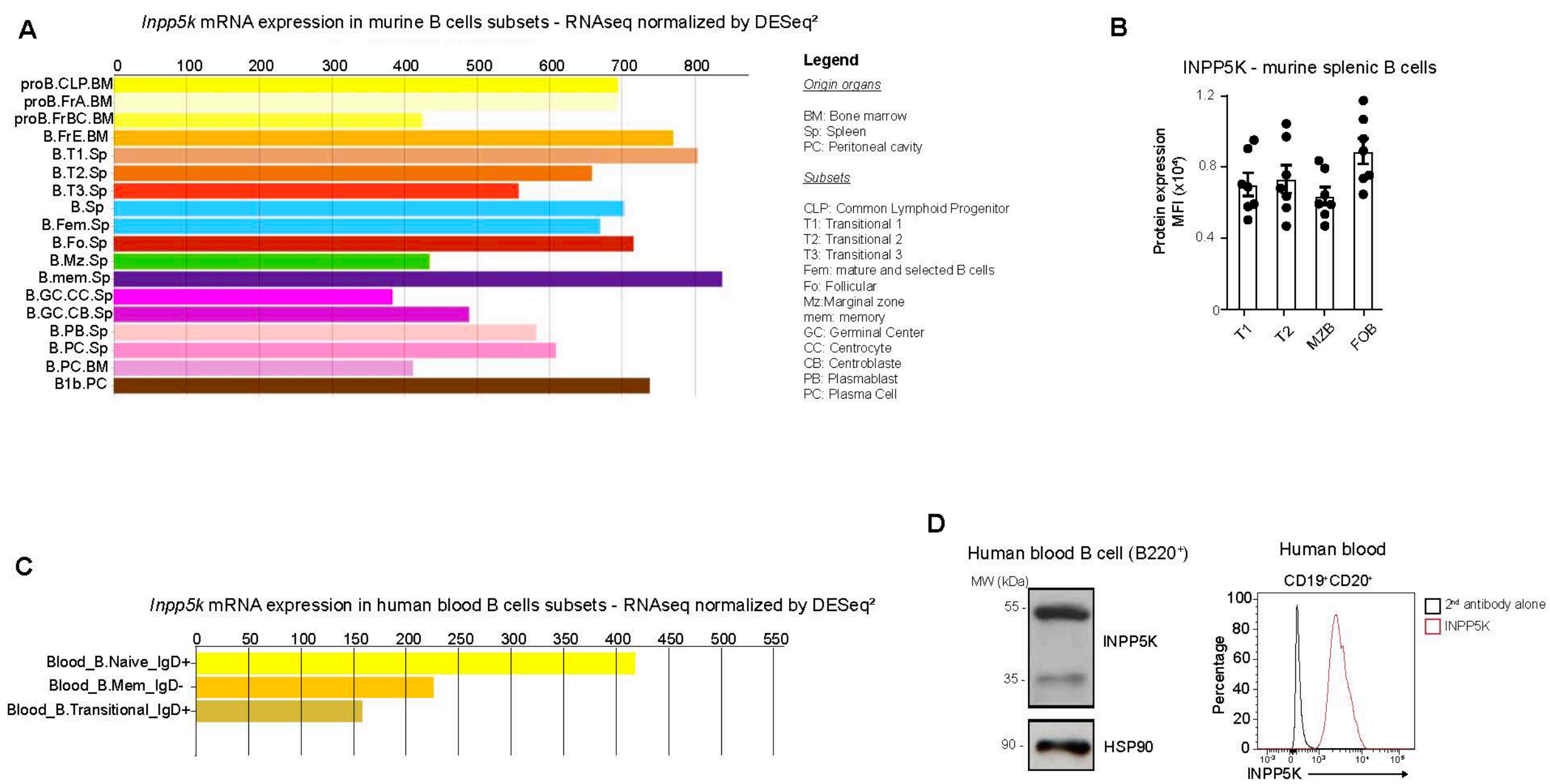

Supplementary Figure 5: INPP5K mRNA and protein expression in mouse and human B cell subsets. RNAseq data on *Inpp5k* mRNA expression in different B cell subsets in mouse (**A**) and human (**C**) were obtained from ImmGen, the Immunological Genome Project (http://www.immgen.org/). **B**. Levels of INPP5K protein were analyzed by flow cytometry in splenic T1, T2, FO and MZ B cell subsets of WT mice. Results represent individual mice and means ± SEM of the mean fluorescence intensity (MFI). n= 6 mice per group. Data are pooled from 2 independent experiments. **D**. The human INPP5K protein was detected in purified human blood B220$^+$ B cells by Western blot (left panels) as well as in CD19$^+$CD20$^+$ B cells by flow cytometry (right panel).

supplementary Figure 6

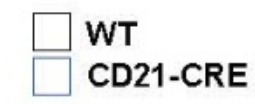

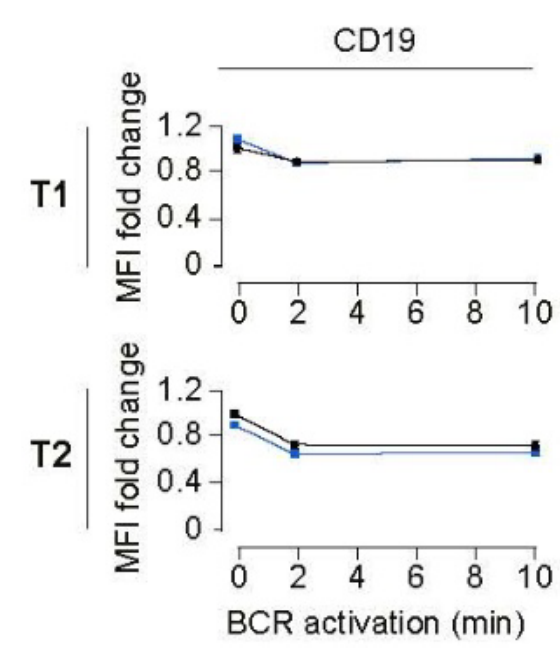

Supplementary Figure 6: Normal CD19 surface expression in CD21-CRE B cells.

Levels of cell surface CD19 were analyzed by flow cytometry in splenic T1 and T2 B cell subsets of WT (black lines) and CD21-CRE (blue lines) mice before (0), 2 or 10 minutes after BCR cross linking. Quantified expression normalized to WT MFI at t=0 (before BCR cross linking) is presented. Results represent means ± SEM. n=11-12 mice per group. Data are pooled from 3 independent experiments.

supplementary Figure 7

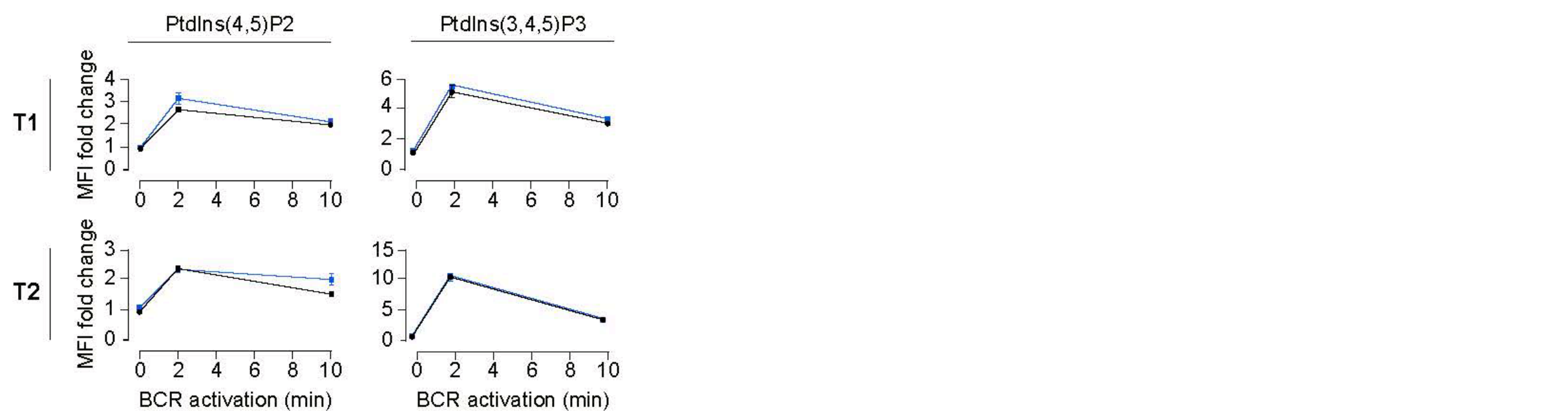

Supplementary Figure 7: Total cellular levels of PtdIns(4,5)P2 and PtdIns(3,4,5)P3 are normal in CD21-CRE B cells. PtdIns(4,5)P2 and PtdIns(3,4,5)P3 levels were analyzed by flow cytometry in splenic T1 and T2 B cell subsets of WT (black lines) and CD21-CRE (blue lines) mice before (0), 2, and 10 minutes after *ex-vivo* BCR cross linking at 37◦C. Quantified expression normalized to WT MFI at t=0 (before BCR cross linking) is presented. Results represent means ± SEM. n= 7 mice per group. Data are pooled from 2 independent experiments.

supplementary Figure 8

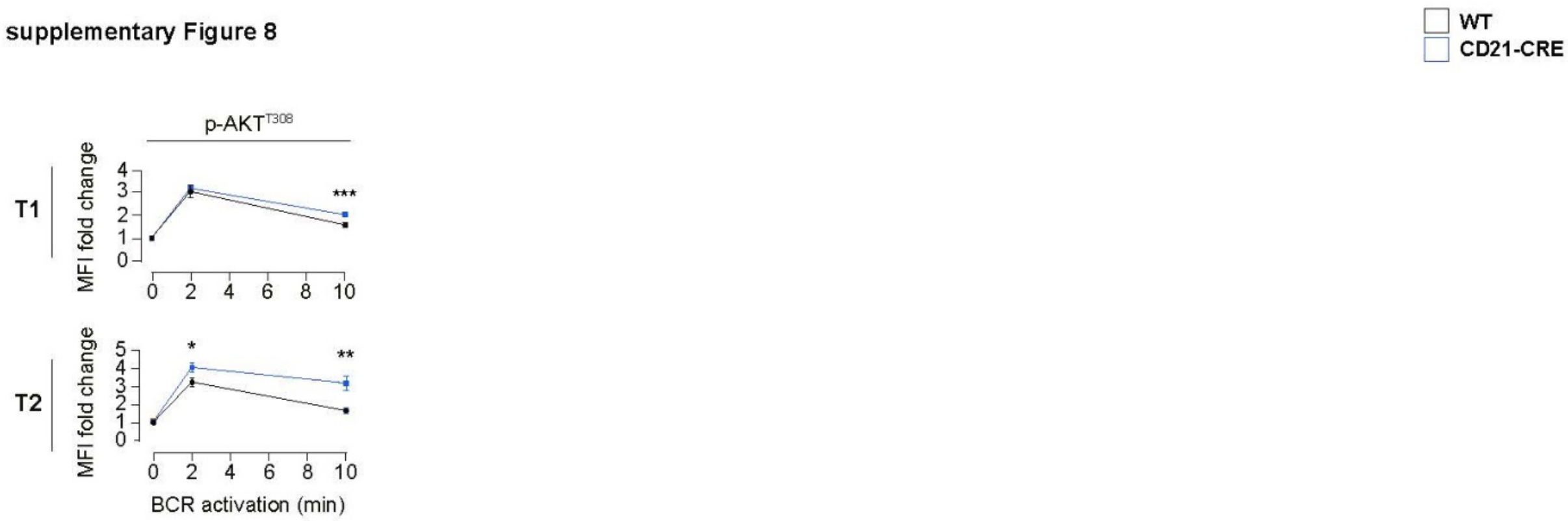

Supplementary Figure 8: Analysis of p-AKT^{T308} in T1 and T2 B cells. Flow cytometry analysis of p-AKT^{T308} in plasma membrane-permeabilized T1 and T2 cells isolated from WT (black lines) and CD21-CRE (blue lines) spleen, before (0), and 2 or 10 minutes after BCR cross linking. Quantified expression normalized to WT MFI at t = 0 (before BCR cross linking) are presented; n= 6 mice per group. Data are pooled from 2 independent experiments. *P* values were calculated using unpaired nonparametric *t* test. *: *P*<0,05; **: *P*<0,01; ***: *P*<0,001.

supplementary Figure 9

A

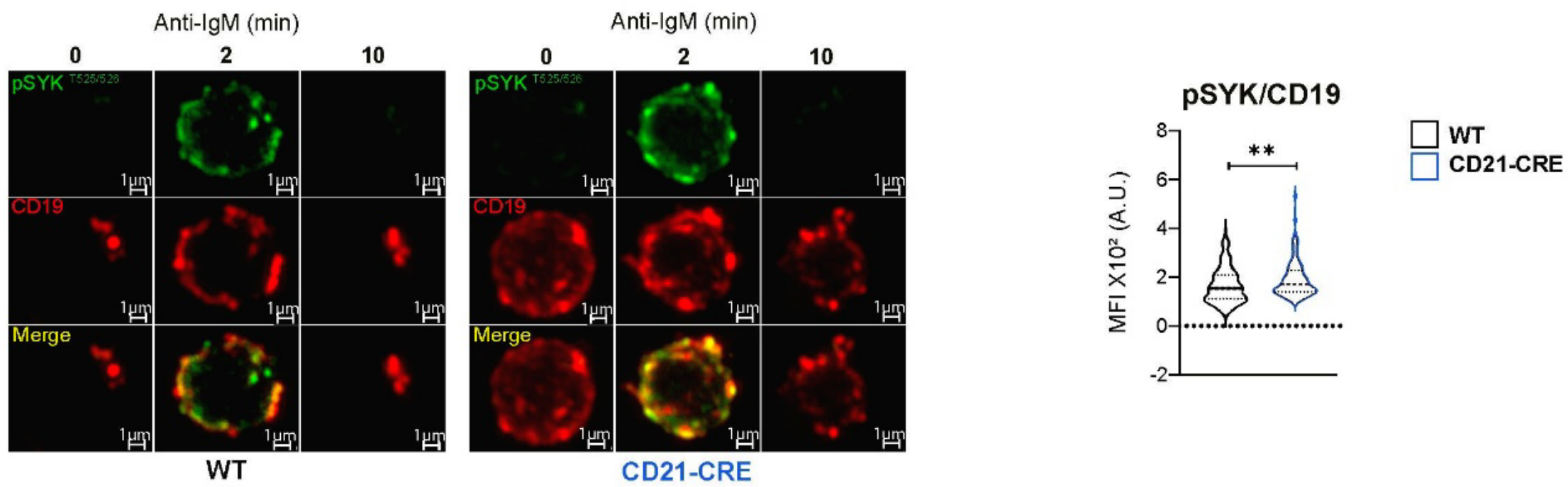

Supplementary Figure 9: Analysis of p-SYK specifically in CD19 microclusters.

Left: representative confocal microscopy images (100X) of splenic T1/T2 B cells sorted from WT and CD21-CRE mice before (0), and 2 or 10 min after BCR cross linking. The CD19 (red) antibody was added to cells before plasma membrane-permeabilization while the p-SYK$^{T525/526}$ (green) antibody was added after membrane permeabilization. White scale bars: 1 µm. Right graph: mean fluorescence intensity (MFI, arbitrary units) of the pSYK$^{T525/526}$ signal colocalized with the CD19 signal in WT (black line) and CD21-CRE (blue line) T1/T2 B cells 2 min after BCR cross linking. No p-SYK$^{T525/526}$ signal was detected before and 10 min after BCR cross linking. Results represent means ± SEM (n=30 cells analyzed per group, from 3 mice). P values were calculated using unpaired nonparametric t test. **: P<0,01.

supplementary Figure 10

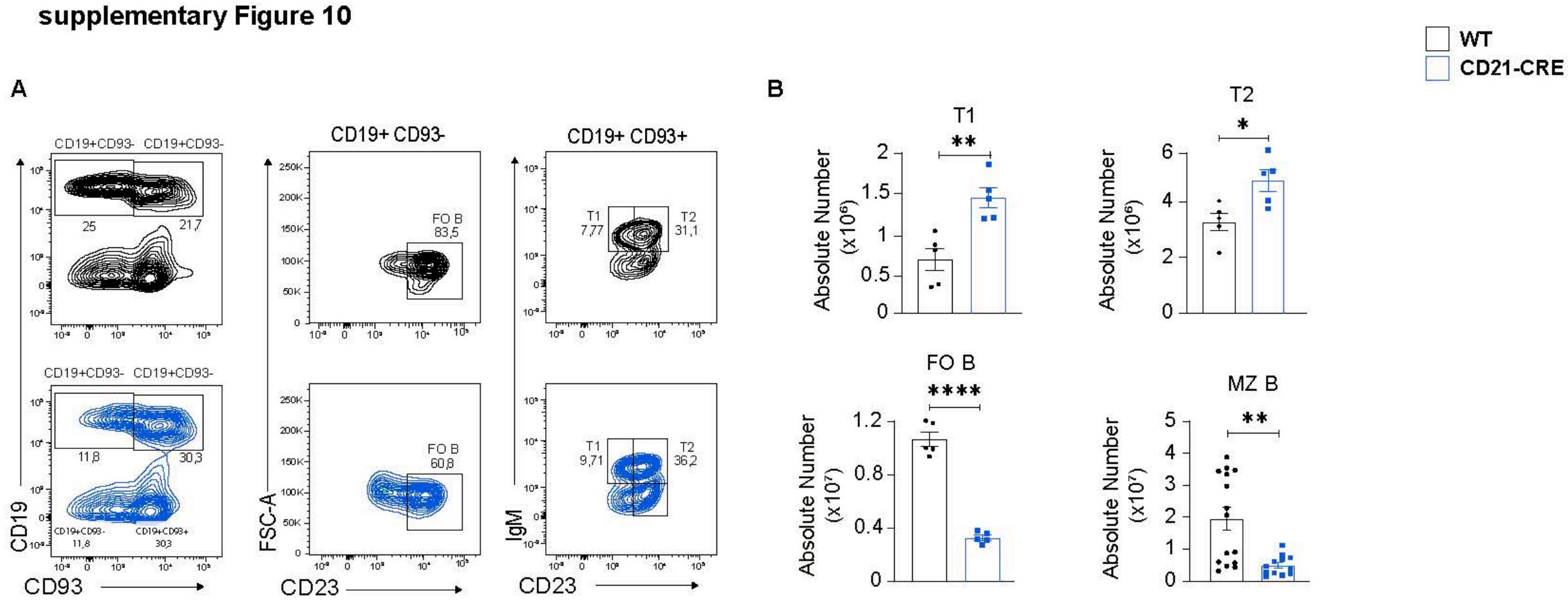

Supplementary Figure 10: An alternative staining strategy to identify splenic B cell subsets. A. An alternative staining strategy was used to identify splenic T1 (CD19^{+}CD93^{+}IgM^{high}CD23^{-}), T2 (CD19^{+}CD93^{+}IgM^{high}CD23^{+}) and FO (CD19^{+}CD93^{-}CD23^{+}) B cells. **B.** B cell numbers in T1, T2, FO and MZ (CD19^{+}IgM^{+}CD23^{-}CD21^{+}) subsets of WT (black columns and dots) and CD21-CRE (blue columns and dots) mice, using the alternative strategy. Results represent individual mice and means ± SEM. n=6-15 mice per group. *P* values were calculated using unpaired nonparametric *t* test. *: *P*<0,05; **: *P*<0,01; ****: *P*<0,0001.

supplementary Figure 11

Inpp5k mRNA expression in human naive B cells

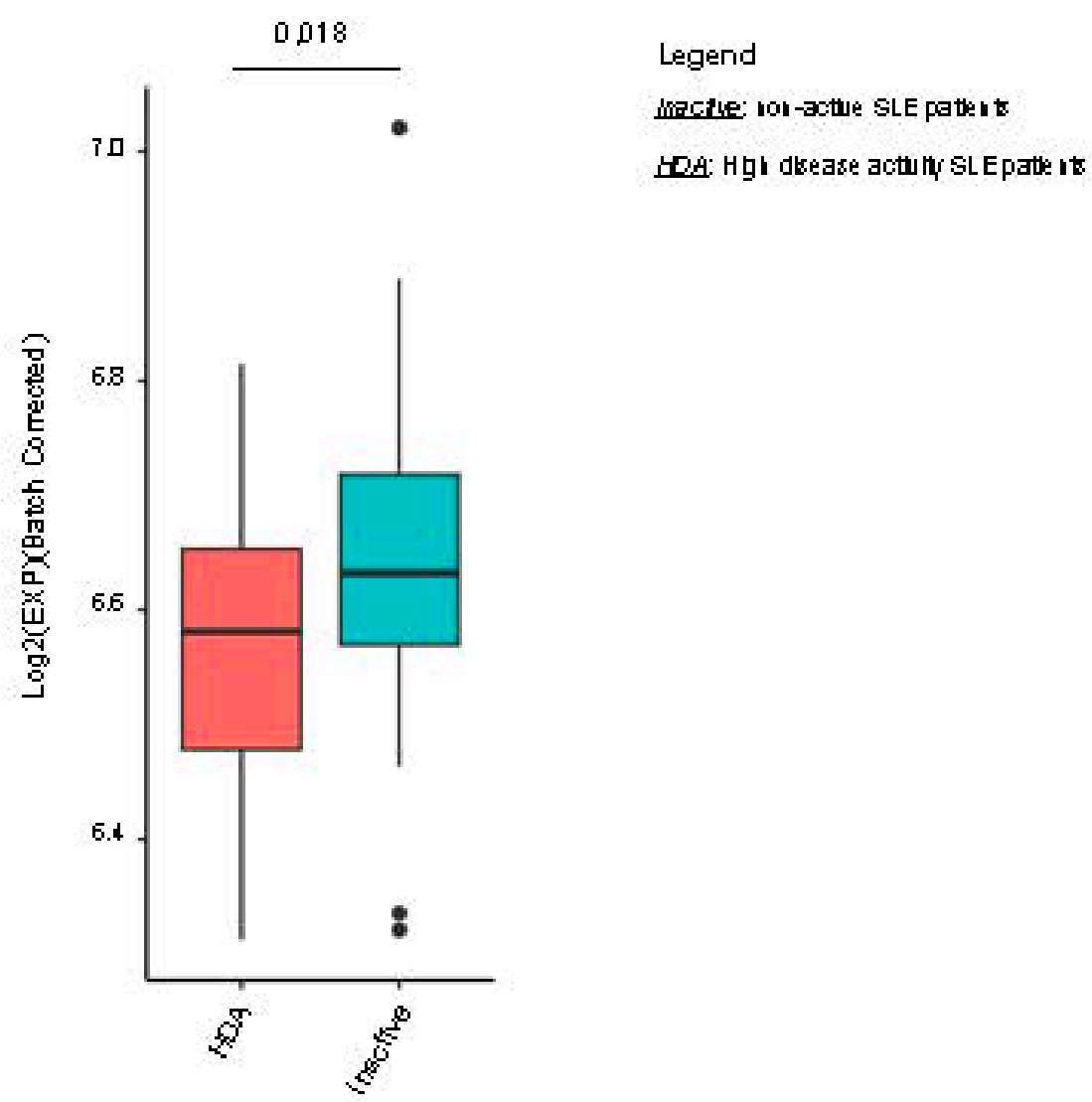

Supplementary Figure 11: Decreased INPP5K mRNA levels in naïve B cells from highly active SLE patients, as compared with inactive SLE patients. As described by Nakano et al. (2), the boxplot represents the expression of 5-phosphatase INPP5K mRNA in naive B cells from 27 high disease activity (HDA) SLE patients and 31 inactive SLE patients.

References:

1. The UniProt Consortium. UniProt: the universal protein knowledgebase in 2025. *Nucleic Acids Res*. **53**, gkae1010 (2024).

2. Nakano, M., Ota, M., Takeshima, Y., Iwasaki, Y., Hatano, H., Nagafuchi, Y., Itamiya, T., Maeda, J., Yoshida, R., Yamada, S., Nishiwaki, A., Takahashi, H., Takahashi, H., Akutsu, Y., Kusuda, T., Suetsugu, H., Liu, L., Kim, K., Yin, X., Bang, S.-Y., Cui, Y., Lee, H.-S., Shoda, H., Zhang, X., Bae, S.-C., Terao, C., Yamamoto, K., Okamura, T.,

Ishigaki, K., & Fujio, K. Distinct transcriptome architectures underlying lupus establishment and exacerbation. *Cell* **185**, 3375-3389 (2022).
