# Supplementary material for: INPP5K limits PtdIns (4,5)P2 accumulation in CD19 microclusters and restrains CD19 co-receptor membrane proximity and BCR-proximal signaling in B cells
Source: Front Immunol. 2026 Sep 3;17:1934292. doi: 10.3389/fimmu.2026.1934292 (PMC13581894; doi:10.3389/fimmu.2026.1934292)
Supplement: Supplementary file 3 [file DataSheet3.pdf]

==

SUPERPLOT GENERATION PIPELINE — METHODOLOGY

CD19 / INPP5K single-cell imaging quantifications

(FRET index, CD19 microcluster volume, BCR microcluster, PtdIns(4,5)P2 MFI,

PtdIns(4,5)P2 density per um3 of CD19)

Author: B. Moes | Last updated: 2026

==

----

1. PURPOSE

This document describes the standard pipeline used to visualize and compare per-cell imaging measurements between genotypes (WT vs B-INPP5K-KD) across timepoints of BCR engagement, using SuperPlots (Lord et al., J Cell Biol 2020;219:e202001064). The pipeline is identical for every SuperPlot in the study; only the input column and the y-axis label change.

----

2. SOFTWARE ENVIRONMENT

- Python 3.10
- openpyxl (read .xlsx)
- NumPy (arrays, medians)
- SciPy (scipy.stats.mannwhitneyu)
- matplotlib (violin + scatter rendering; pdf.fonttype=42, svg.fonttype='none')
- Fonts: Arial (fallback: Liberation Sans, metric-identical; then DejaVu Sans)

Install:

```
pip install openpyxl numpy scipy matplotlib
```

----

3. EXPERIMENTAL DESIGN AND REPLICATE LOGIC (READ FIRST)

- Cells were POOLED from 3 mice per genotype per experiment (limited cell yield).
- The individual mouse is therefore NOT recoverable as a statistical unit.
- The INDEPENDENT EXPERIMENT is treated as the biological replicate (n = 2).
- Consequence for statistics:
  * The large symbols in each SuperPlot are the PER-EXPERIMENT MEDIANS (the replicate-level summary); their concordance in direction across the two experiments is the primary evidence of reproducibility.
  * Any per-cell test (Mann-Whitney U on pooled cells) is PSEUDOREPLICATED and is reported ONLY as a descriptive comparison, never as the primary inferential test. With n = 2 experiments a formal replicate-level test is not powered; reproducibility is judged from the two per-experiment medians.

----

4. INPUT DATA FORMAT (.xlsx, wide format)

- One sheet, wide layout.
- Header rows identify EXP block, timepoint (0/2/10 min) and genotype (WT / CD21-CRE, where CD21-CRE = B-INPP5K-KD).
- Each column = one condition; each row below the header = one cell (one value).
- Column maps used in this study (0-based column indices):
  * MFI files (PIP2_CD19.xlsx):
    EXP1: t0 WT=1 KD=2 | t2 WT=3 KD=4 | t10 WT=5 KD=6
    EXP2: t0 WT=9 KD=10| t2 WT=11 KD=12| t10 WT=13 KD=14
  * Volume-normalized file (PIP2_CD19_volume_1.xlsx, sheet 'Feuil2', data row>=5):
    EXP1: t0 WT=0 KD=1 | t2 WT=2 KD=3 | t10 WT=4 KD=5
    EXP2: t0 WT=8 KD=9 | t2 WT=10 KD=11| t10 WT=12 KD=13

  (Adjust the column map to match each file; verify header labels every time.)

----

5. QUANTIFICATION SOURCE (upstream of this pipeline)

Per-cell values are exported upstream and are metric-specific:

- FRET normalized index: ImageJ/Fiji macro (Bio-Formats import), donor/acceptor
  single-label controls define compensation coefficients
  Coef_B = I_DAb/I_DD (donor bleed-through), Coef_D = I_DA/I_AA (acceptor
  cross-excitation); per cell:

      FRET_corr = I_DA - Coef_B*I_DD - Coef_D*I_AA
      index   = FRET_corr / I_DD   (may exceed 1; not a physical efficiency)

  Cell mask from DAPI (Gaussian blur, Huang threshold, dilate, fill holes,
  watershed, Analyze Particles).
- CD19 / BCR microcluster volume and PtdIns(4,5)P2 density: Airyscan imaging,
  Imaris Surfaces segmentation (background subtraction, threshold, min volume
  0.3 um3); PtdIns(4,5)P2 density = PtdIns(4,5)P2 fluorescence within each CD19
  surface divided by the CD19 surface volume (MFI per um3).
  >>> Record the exact instrument, Imaris version and thresholds in Methods. <<<

----

6. STATISTICS

- Per timepoint and genotype pair: two-sided Mann-Whitney U test
  (scipy.stats.mannwhitneyu, alternative='two-sided') on pooled per-cell values.
- Reported as DESCRIPTIVE only (see section 3). Report alongside the two
  per-experiment medians and the KD/WT median ratio per experiment.
- Significance glyphs: * p<0.05 ** p<0.01 *** p<0.001 **** p<0.0001 ns p>=0.05
- Effect direction is assessed as the sign of (KD median - WT median) within EACH
  experiment; an effect is called reproducible only if both experiments agree.

----

7. PLOTTING CONVENTIONS (identical across all SuperPlots)

- Y-axis: log10 scale (single-cell distributions are right-skewed).
- Violin: white fill, coloured edge by genotype, full opacity, no extrema bars.
- Individual cells: small points (s=5), coloured by EXPERIMENT (Exp1 #777777 grey, Exp2 #c48a1a orange), alpha 0.30, horizontally jittered.
- Per-experiment median: large point (s=70), face = experiment colour, edge = genotype colour.
- Genotype colours: WT = #000000 (black), B-INPP5K-KD = #235BD1 (blue).
- X positions: WT left, KD right within each timepoint cluster (0/2/10 min).
- Significance bracket + glyph drawn above each WT/KD pair.
- Export: PDF (vector, fonttype 42) + PNG (300 dpi). Keep text as text (svg.fonttype='none') for downstream editing.

----

8. HOW TO RUN

----

1) Place the .xlsx next to the script (or edit SRC).
2) Set the sheet name, the data start row, and the column map to match the file.
3) Set the y-axis label to the metric (e.g. "CD19 microcluster volume (um3)", "PtdIns(4,5)P2 MFI in CD19 clusters", "PtdIns(4,5)P2 MFI / um3 CD19 signal").
4) Run: python3 <script>.py
5) Outputs: <name>.pdf and <name>.png, plus a console table of per-timepoint WT/KD medians, KD/WT ratio and descriptive p-value.

----

9. REPRODUCIBILITY NOTES / THINGS TO VERIFY EACH TIME

- Re-read the header row: confirm which column block is EXP1 vs EXP2 (label typos have occurred, e.g. two "EXP1" headers).
- Confirm timepoint order and WT/KD assignment before trusting the plot.
- State in Methods whether compensation coefficients (FRET) were determined once or independently per experiment — only claim per-experiment if true.

- Do not report per-cell p-values as inferential; keep them descriptive.

- One value = one cell; cells pooled from 3 mice/genotype/experiment; n = « exp.

==
